# Three-dimensional distribution of cortical synapses: a replicated point pattern-based analysis

**DOI:** 10.3389/fnana.2014.00085

**Published:** 2014-08-26

**Authors:** Laura Anton-Sanchez, Concha Bielza, Angel Merchán-Pérez, José-Rodrigo Rodríguez, Javier DeFelipe, Pedro Larrañaga

**Affiliations:** ^1^Departamento de Inteligencia Artificial, Escuela Técnica Superior de Ingenieros Informáticos, Universidad Politécnica de MadridMadrid, Spain; ^2^Laboratorio Cajal de Circuitos Corticales, Centro de Tecnología Biomédica, Universidad Politécnica de MadridMadrid, Spain; ^3^Departamento de Arquitectura y Tecnología de Sistemas Informáticos, Escuela Técnica Superior de Ingenieros Informáticos, Universidad Politécnica de MadridMadrid, Spain; ^4^Instituto Cajal, Consejo Superior de Investigaciones CientíficasMadrid, Spain

**Keywords:** spatial distribution of synapses, neocortex, dual-beam electron microscopy, FIB/SEM, replicated spatial point patterns, random sequential adsorption, 3D Ripley's *K* function, Besag's *L* function

## Abstract

The biggest problem when analyzing the brain is that its synaptic connections are extremely complex. Generally, the billions of neurons making up the brain exchange information through two types of highly specialized structures: chemical synapses (the vast majority) and so-called gap junctions (a substrate of one class of electrical synapse). Here we are interested in exploring the three-dimensional spatial distribution of chemical synapses in the cerebral cortex. Recent research has showed that the three-dimensional spatial distribution of synapses in layer III of the neocortex can be modeled by a random sequential adsorption (RSA) point process, i.e., synapses are distributed in space almost randomly, with the only constraint that they cannot overlap. In this study we hypothesize that RSA processes can also explain the distribution of synapses in all cortical layers. We also investigate whether there are differences in both the synaptic density and spatial distribution of synapses between layers. Using combined focused ion beam milling and scanning electron microscopy (FIB/SEM), we obtained three-dimensional samples from the six layers of the rat somatosensory cortex and identified and reconstructed the synaptic junctions. A total volume of tissue of approximately 4500μm^3^ and around 4000 synapses from three different animals were analyzed. Different samples, layers and/or animals were aggregated and compared using RSA replicated spatial point processes. The results showed no significant differences in the synaptic distribution across the different rats used in the study. We found that RSA processes described the spatial distribution of synapses in all samples of each layer. We also found that the synaptic distribution in layers II to VI conforms to a common underlying RSA process with different densities per layer. Interestingly, the results showed that synapses in layer I had a slightly different spatial distribution from the other layers.

## 1. Introduction

A very dense network of neuronal and glial processes occupies the space between the cell bodies of the neurons, glia, and blood vessels. This is commonly referred to as “the neuropil.” Given that most synapses are found here and the neuropil accounts for the largest volume of the cerebral cortex, it follows that most synaptic interactions take place in the neuropil (Alonso-Nanclares et al., 2008). The majority of these synapses are chemical synapses (for simplicity's sake referred to as synapses) which are identified at the electron microscope level for the following elements: synaptic vesicles in the presynaptic axon terminal adjacent to the presynaptic density, a synaptic cleft (with electron-dense material in the cleft) and densities on the cytoplasmic faces in the pre- and postsynaptic membranes.

One major issue in cortical circuitry is to ascertain how synapses are distributed and whether or not synaptic connections are specific or not (DeFelipe et al., 2002b). To understand the anatomical design principles of cortical circuits, it is essential to analyze the ultrastructure of all components of the neuropil and in particular the number and spatial distribution of synapses. Furthermore, synaptic size plays an important role in the functional properties of synapses (Schikorski and Stevens, 1997; Takumi et al., 1999; Lüscher et al., 2000; Tarusawa et al., 2009). Thus, numerous researchers have been trying to find simple and accurate methods for estimating the distribution, size and number of synapses. To this end, two sampling procedures are currently available: one is based on serial reconstructions and the other on single sections. Clearly, serial reconstruction should be the method of choice for the challenging task of unraveling the extraordinary complexity of the nervous system. Indeed, serial sectioning transmission electron microscopy is a well-established and mature technology for collecting three-dimensional data from ultrathin sections of brain tissue (Stevens et al., 1980; Harris et al., 2006; Hoffpauir et al., 2007; Mishchenko et al., 2010; Bock et al., 2011). It is based on imaging ribbons of consecutive sections with a conventional transmission electron microscope (TEM). However, the major limitation is that it is extremely time-consuming and difficult to obtain long series of ultrathin sections, often making it impossible to reconstruct large volumes of tissue. Hence, the recent development of automated electron microscopy techniques is a vital step forward in the study of neuronal circuits (Briggman and Denk, 2006; Knott et al., 2008; Merchán-Pérez et al., 2009). Using combined focused ion beam (FIB) milling and scanning electron microscopy (SEM), we have recently shown (Merchán-Pérez et al., 2014) that synapses in the neuropil of layer III of the rat somatosensory cortex show a nearly random spatial distribution, with the only constraint that they cannot overlap in space; distribution that can be modeled by a random sequential adsorption (RSA) process (Evans, 1993), where synapses are given a random position in space and assigned a certain size derived from experimental data.

The aim of this research was to explore the three-dimensional distribution of synapses in the cerebral cortex as a whole and, particularly, find out whether there is a general pattern of distribution of synapses for the six cortical layers, and identifying any possible similarities and differences between layers. To do this, we studied the density of synapses and their spatial distribution as follows. First, we analyzed the synaptic density in each of the six layers of the somatosensory cortex and examined whether there were significant differences between layers. Second, we performed spatial modeling to test whether each sample from different neocortical layers conforms to an RSA model. Third, we used replicated spatial point patterns to analyze similarities and differences in the synaptic spatial distribution between groups of samples of each cortical layer.

Finally, note that we have used postnatal day 14 Wistar rats since we intend to integrate these data with other anatomical, molecular and physiological data that have already been collected from the same cortical region of the P-14 Wistar rats. The final goal is to create a detailed, biologically accurate model of the brain within the framework of the Blue Brain Project (http://bluebrain.epfl.ch/).

## 2. Materials and methods

### 2.1. Tissue preparation and three-dimensional electron microscopy

Three male Wistar rats sacrificed on postnatal day 14 were used for this study. They were handled in accordance with the guidelines for animal research set out in European Union Directive 2010/63/EU, and all procedures were approved by the Spanish National Research Council (CSIC) local ethics committee. Animals were administered a lethal intraperitoneal injection of sodium pentobarbital (40 mg/kg) and were intracardially perfused with 2% paraformaldehyde and 2.5% glutaraldehyde in 0.1M phosphate buffer. The brain was then extracted from the skull and vibratome sections (150 microns thick) were obtained, processed for electron microscopy and flat-embedded in Araldite according to a previously described protocol (Merchán-Pérez et al., 2009, 2014). Three-dimensional brain tissue samples were obtained from flat-embedded vibratome sections using a combined focused ion beam/scanning electron microscope (FIB-SEM). This electron microscope (Neon40 EsB, Carl Zeiss NTS GmbH, Oberkochen, Germany) combines a high-resolution field emission SEM column with a focused gallium ion beam which mills the sample surface, removing thin layers of material on a nanometer scale.

Stacks of serial sections were obtained from the six cortical layers (see Table 1). Samples from layer III were used in a previous study (Merchán-Pérez et al., 2014). To select the exact location of the samples in the different cortical layers, we first obtained plastic semithin sections (2 μm thick) from the block surface, which we stained with toluidine blue. These sections were then photographed with a light microscope. The last of these light microscope images (corresponding to the section immediately adjacent to the block face) was then collated with low power SEM photographs of the block surface. In this way, we were able to accurately identify the regions of the neuropil to be studied. To obtain each sample from the selected location, the FIB was positioned perpendicular to the block surface. Next, a trench (approximately 30 μm wide, 20 μm high and 15 μm deep) was excavated on the block surface. Since the SEM column is positioned at an angle of 54° to the FIB column, the distal face of the trench can be imaged with the SEM after each milling cycle. The milling/imaging cycle was then set to remove 20 nm of material from the distal face of the trench. After removing each slice, the milling process was paused and the freshly exposed surface was imaged with a 1.8 kV acceleration potential using the in-column energy selective backscattered electron detector (EsB). The milling and imaging processes were sequentially repeated to acquire long series of images by means of a fully automated procedure, outputting a stack of images that represented a three-dimensional sample of the tissue. The total number of serial sections per sample ranged from 189 to 363 (mean = 258.6), the imaged field of view was approximately 7.6 × 5.7 microns, and image resolution in the XY plane ranged from 3.7 to 11.10 nm/pixel. Z-axis resolution (section thickness) was 20 nm. In this way, the total tissue volume that was actually milled away during the milling/imaging cycles was relatively small, and we were able to obtain multiple samples from different layers or from neighboring regions within the same layer.

**Table 1:** **Animal ID, volume, counts, and density of synaptic junctions per sample in each layer of the somatosensory cortex**.

	**Sample**	**Animal**	**Volume (μm^3^)**	**No. of synapses**	**synapses/μm^3^**
Layer I
	1	w33	210.61	180	0.855
	2	w35	177.20	128	0.722
Layer II
	1	w33	224.35	230	1.025
	2	w35	139.51	127	0.910
	3	w35	149.03	206	1.382
Layer III
	1	w31	149.13	147	0.986
	2	w31	157.15	109	0.694
	3	w33	186.45	173	0.928
	4	w33	176.44	178	1.009
	5	w33	176.28	167	0.947
	6	w33	175.55	165	0.940
	7	w33	191.28	189	0.988
	8	w35	247.58	198	0.800
	9	w35	178.40	201	1.127
	10	w35	165.06	168	1.018
Layer IV
	1	w33	154.59	172	1.113
	2	w35	140.63	178	1.266
	3	w35	123.81	162	1.308
Layer V
	1	w33	165.62	117	0.706
	2	w33	218.01	198	0.908
	3	w33	207.95	175	0.842
Layer VI
	1	w33	185.32	92	0.496
	2	w35	183.55	85	0.463
	3	w31	179.97	102	0.567
	4	w31	280.09	107	0.382
		All Samples	4543.55	3954	0.870
Mean	
		Layer I	193.91	154	0.794
		Layer II	170.96	188	1.098
		Layer III	180.33	170	0.940
		Layer IV	139.68	171	1.222
		Layer V	197.19	163	0.828
		Layer VI	207.23	97	0.466

*Total quantities and mean for each layer are shown*.

Synaptic junctions within each stack of serial sections were visualized, automatically segmented and reconstructed in three dimensions using Espina software (Morales et al., 2011). In order to calculate the number of synapses per unit volume, we applied a three-dimensional unbiased counting frame (Howard and Reed, 2005). Espina software output the volume of the unbiased counting frame, the number of synaptic junctions inside the frame, the spatial position of the centroids or centers of gravity of the synaptic junctions, and an estimation of their sizes using Feret's diameter (the diameter of the smallest sphere circumscribing the synaptic junction). Brain tissue shrinks during processing for electron microscopy, especially during osmication and plastic embedding. To estimate the shrinkage in our samples, we measured the surface area and thickness of the vibratome sections before and after they were processed for electron microscopy (Oorschot et al., 1991; Merchán-Pérez et al., 2009). The estimated linear, area and volume shrinkage factors were 0.90, 0.81, and 0.73, respectively. To obtain an estimate of the pre-processing values, all measured distances, areas and volumes were divided by their corresponding shrinkage factor. After correcting for tissue shrinkage, the samples that were subsequently used for spatial statistical analysis consisted of a cloud of points representing the centers of gravity or centroids of synaptic junctions. Each of these points had an associated Feret's diameter as an estimation of the size of each synaptic junction.

### 2.2. Spatial statistics

Within the field of spatial statistics, spatial point processes are mathematical models that describe the arrangement of elements randomly or irregularly distributed in space. A spatial point pattern is defined as a realization of a spatial point process (Illian et al. (2008) provides a good introduction to the topic). The elements in the pattern are represented by point coordinates in the appropriate dimension. In this study, our elements are synaptic junctions located in three dimensions.

Spatial point process statistics provides the tools to characterize patterns in terms of the number and distribution of the elements. To do this, two aspects are mainly analyzed: intensity (average number of points per unit volume, denoted by λ) and inter-point interactions, closely related to distances between points.

#### 2.2.1. Synapse density in different layers

The most important numerical summary characteristic for a point process is the intensity λ. Point intensity is the simplest distributional property and is similar to the use of the sample mean in classical statistics. Thus, the first step in our analysis was to estimate the synaptic density of each layer and, more specifically, to study whether there were significant differences between synaptic densities in different layers of the somatosensory cortex. We used the simulation process described below along with a multiple mean comparison test.

**Figure 1:**
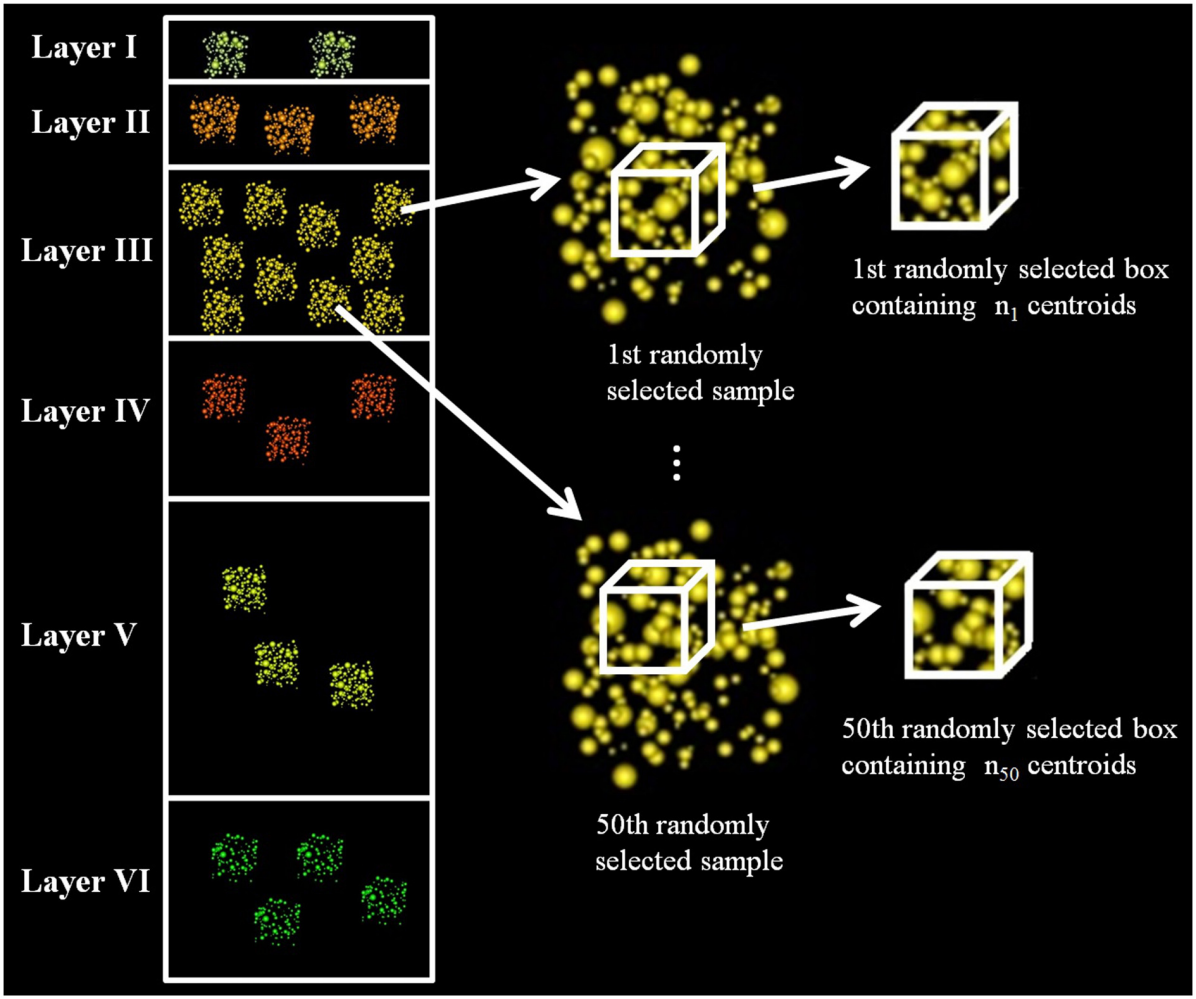
**Diagram of data extraction to analyze whether the synaptic densities of cortical layers are significantly different**. The figure shows how we randomly selected a sample from layer III, then we extracted, also randomly, a box inside this sample and counted the number of synaptic junctions in the box. We repeated this process 50 times for each layer. The dimensions of the box were the same for all layers, and it had the maximum volume that could be extracted from all the samples, i.e., it had the minimum length in each dimension (x, y, z) considering all samples.

We calculated a fixed-volume sampling box to extract subsamples from the original experimental samples. The x, y, z dimensions of this box were equal to the smallest x, y, z dimensions of the experimental samples, so the box could be applied to any of the samples without exceeding their boundaries. We then used this box to extract centroids from randomly selected samples of each layer at random locations. We repeated this process 50 times for each layer, thus obtaining 50 different synaptic densities per layer. See Figure 1.

To study whether there were significant differences between synaptic densities of the different layers, we performed a multiple mean comparison test on the 50 extracted densities for each of the six cortical layers. Because not all of the necessary assumptions for ANOVA were satisfied (data were normally distributed but homoscedasticity was not met, i.e., the variance of data in each layer was not the same), we used the Kruskal-Wallis test and then applied the Mann-Whitney test with the Bonferroni method to adjust the *p*-values for pair-wise comparisons.

#### 2.2.2. Modeling of spatial point processes

Merchán-Pérez et al. (2014) recently showed that the RSA model adequately describes the spatial distribution of synaptic junctions in layer III. The second step in the analysis of the entire cerebral cortex was to test the RSA model for each of our samples from layer I to VI.

Although virtually all cortical synapses can be accurately identified as asymmetric (or Gray's type I) and symmetric (or Gray's type II) using FIB/SEM (Merchán-Pérez et al., 2009), we considered synaptic junctions as a whole. This was because it was not feasible to test RSA models for such a small number of symmetric synapses (they accounted for less than 10% of the total number of synapses found in any cortical layer). Furthermore, as reported previously (Merchán-Pérez et al., 2014), results were similar when all synapses (asymmetric and symmetric) were studied as a single group and when only asymmetric synapses were analyzed. Thus, for simplicity's sake, we will use synaptic junctions to refer to both types of synapses.

An RSA process (Evans, 1993) is a type of hard-core process, i.e., two points cannot be placed closer than a minimum distance, where locations are chosen randomly, subject only to the distance constraint. These minimum distances can be fixed or, as in our case, calculated according to a probability density function. Considering that the synaptic junctions cannot overlap, and therefore the minimum distances between synapses are limited by the size of the junctions at least, the RSA process is particularly well suited here. We have used Feret's diameter of each synaptic junction as an estimate of its size. As in Merchán-Pérez et al. (2014) for layer III, we found that Feret's diameters in all layers were lognormally distributed.

To test the RSA models we used one of the summary characteristics most commonly used in the analysis of spatial point processes, namely Ripley's *K* function and, particularly, a common transformation of it, Besag's *L* function (Ripley, 1977).

Ripley's *K* function for a distance *d*, *K*(*d*), is defined as the expected number of other points of the process within a distance *d* of a typical point of the process divided by the intensity. The Miles-Lantuéjoul-Stoyan-Hanisch translation edge-correction is often used to estimate *K*(*d*) (Ohser, 1983; Baddeley et al., 1993):

(1) $$\hat{K}(d)=\frac{vol{\left(B\right)}^{2}}{N{\left(B\right)}^{2}}\sum_{x_{k}\in B}\sum_{x_{l}\neq x_{k}}\frac{1\{||x_{k}-x_{l}||\leq d\}}{\gamma_{B}(x_{k}-x_{l})},$$

where **1**{·} denotes the indicator function, ‖·‖ is the Euclidean distance, *N*(*B*) is the number of points falling in a region *B*⊂ ℝ^3^, *x_k_*, *k* = 1, …, *N*(*B*) are the observed points, *vol*(*B*) is the volume of the region *B* and γ_*B*_ is the ‘set covariance’, γ_*B*_(*x_k_* − *x_l_*) = vol({*x*|*x* + *x_k_* − *x_l_* ∈ *B*}) = *vol*(*B* ∩ (*B* − (*x_k_* − *x_l_*))).

The homogeneous spatial Poisson point process, also known as complete spatial randomness (CSR), is considered as the reference model in spatial point process statistics, since it represents a boundary condition between regular and clustered patterns. A random pattern, where a point is equally likely to occur at any location regardless of the locations of other points, follows a CSR process. The patterns known as regular patterns show repulsion, i.e., the distances between points are larger than expected in a random pattern of the same intensity. Furthermore, patterns where points tend to be closer than they should be for a given intensity are known as clustered patterns.

The three-dimensional CSR process has the following expression for the *K* function (a clustered pattern curve will be shifted to the left, whereas a regular pattern curve will be shifted to the right):

(2) $$K_{CSR}(d)=\frac{4}{3}\pi d^{3}.$$

Besag's *L* function is a commonly used transformation of the *K* function. The 3D expression is:

(3) $$L(d)=\sqrt[{3}]{\frac{3}{4\pi }K(d)}.$$

This transformation converts the CSR *K* function to the straight line *L_CSR_*(*d*) = *d*, making the plots much easier to assess visually. The transformation approximately stabilizes the variance of the estimator, also facilitating deviation assessment. For the *L* function, a regular pattern curve will be below the diagonal (CSR) and a clustered pattern will be above.

The expression of Ripley's *K* function for the RSA process is analytically unknown, so we have to use RSA simulations. To simulate an RSA process we need to know its intensity and the probability density function of the minimum distances between points. In our case, we need the synaptic density λ and the μ and σ parameters of the lognormal distribution of Feret's diameters. An RSA process simulation starts with an empty window to which spheres, whose radii follow the lognormal distribution fitted using Feret's diameters, are added randomly one at a time. If the new simulated synapse intersects with any existing sphere, the new sphere is rejected, and another sphere is generated with another location and radius. The process continues until the target intensity is reached.

**Figure 2:**
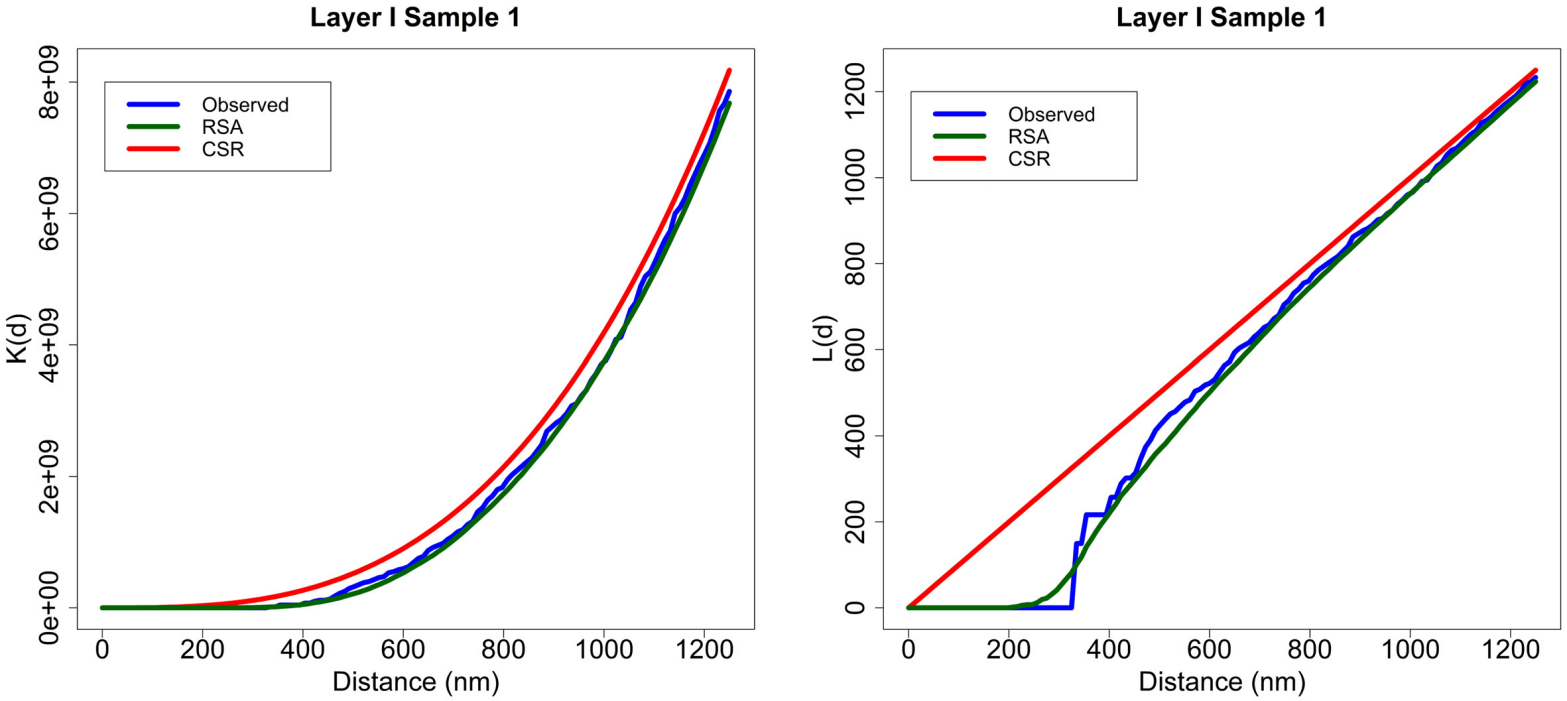
**Layer I, Sample 1**. An example of *K* and *L* functions for CSR and RSA processes. *K* (left) and *L* (right) functions of the experimentally observed data (blue) along with the theoretical CSR (red) and the average of 99 RSA process simulations fitted for sample 1 (green). The *K* functions of the sample, CSR and RSA processes are very similar. The *L* functions of the RSA and the experimentally observed sample are positioned well below the diagonal (CSR) for short distances and are fairly close to the diagonal for larger distances.

For example, Figure 2 shows the *K* and *L* summary functions of experimental sample 1 from Layer I (blue), the average of 99 RSA simulations performed for this sample (green) and the functions for a CSR process (red). Each RSA simulation had the same intensity as the original sample, and the size of simulated synapses was calculated according to the lognormal distribution fitted using Feret's diameters of all the synapses of the sample. Generally, the *K* functions were very similar to each other across all distances for all the samples. Moreover, for short distances (200–300 nm), the *L* functions of the samples and RSA processes were well below the diagonal line (CSR) representing the empty space around centroids which should not contain any centroid (non-overlapping synapse constraint). From about 400 nm onwards, the *L* functions of both models and experimental samples were again very similar to each other.

To test differences between two summary functions we used simulation-based envelopes. The statistical rationale of this common procedure is to be found in Monte Carlo testing. Taking the advice of Baddeley et al. (2014), we transformed the *K* function into the *L* function and used global envelopes since we had no prior information about the range of spatial interaction. Note that Monte Carlo tests “are strictly invalid, and probably conservative, if parameters have been estimated from the data” (Diggle, 2003). To overcome this obstacle, we adjusted an RSA process for each sample *j* in each layer *i* (*i* = I, …, VI) and estimated the parameters $\hat{\lambda }$_*ij*_, $\hat{\mu }$_*ij*_ and $\hat{\sigma }$_*ij*_ using only the remaining samples of the same layer. The sizes of the simulated synapses were calculated according to the lognormal distribution fitted using Feret's diameters of these remaining (*m_i_* − 1) samples in layer *i*, where *m_i_* is the number of samples in layer *i*. If *vol_it_* denotes the volume of sample *t* in layer *i*, then

(4) $${\hat{\lambda }}_{ij}=\frac{\sum_{\begin{array}{c} t=1\\ t\neq j \end{array}}^{m_{i}}\lambda_{it}vol_{it}}{\sum_{\begin{array}{c} t=1\\ t\neq j \end{array}}^{m_{i}}vol_{it}}.$$

**Figure 3:**
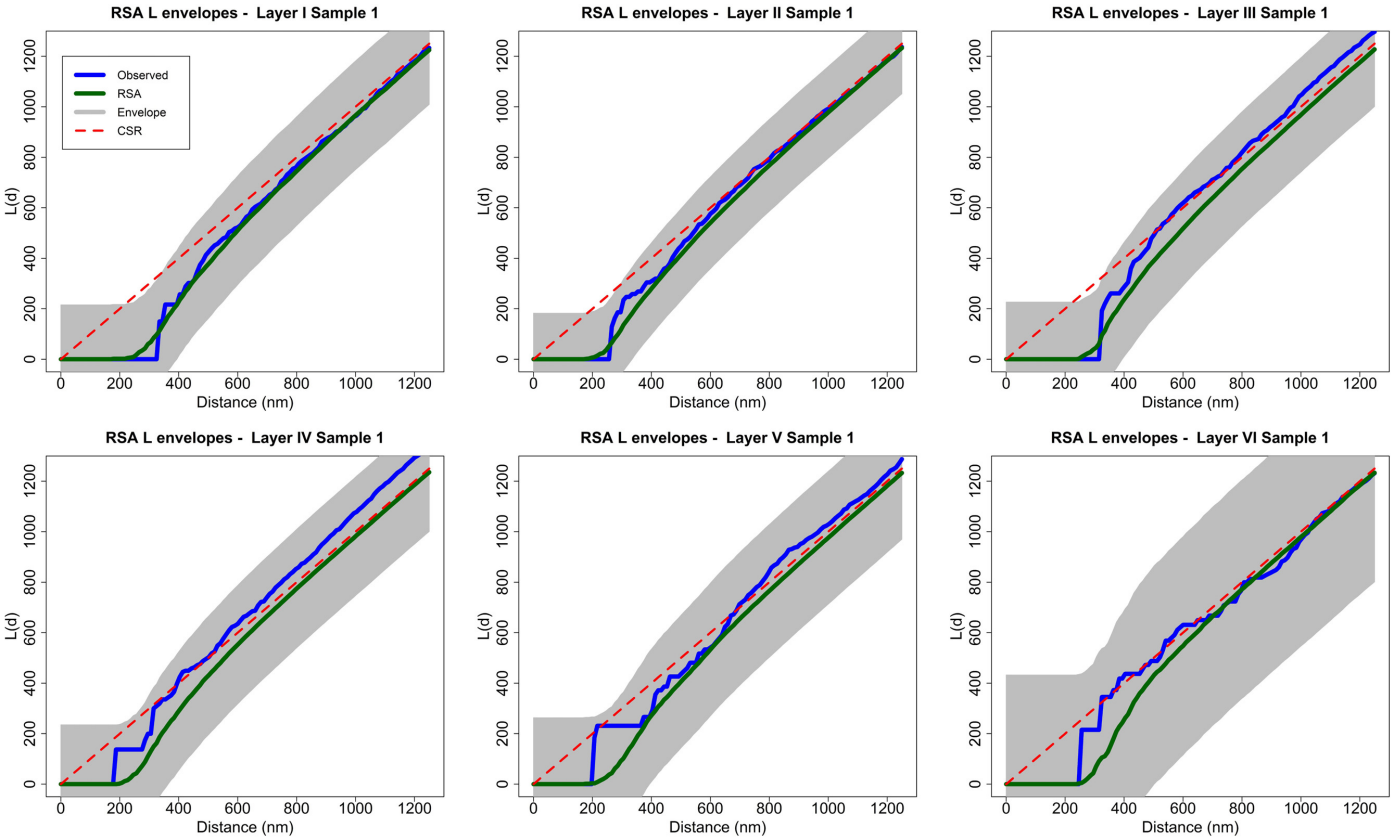
**Analysis of spatial patterns using global envelopes (sample 1 for each layer of the somatosensory cortex)**. The *L* functions of the experimentally observed samples are shown in blue, and the averages of 99 RSA simulations are shown in green. The shaded area represents the envelopes of values calculated from a separate set of 99 RSA simulations. We do not reject the RSA null hypothesis for any sample because no observed *L* function lies outside the envelope for any value of distance *d*. The results for all samples in the study were the same (see Supplementary material). Dashed red lines show the theoretical value for CSR (for the purpose of visual comparison only).

The RSA null hypothesis was tested as follows. For each sample, we performed 99 RSA simulations with the described parameters. We calculated the average *L* function of all these simulations and took this average, *L*, to be an estimate of the theoretical mean value of the *L* summary statistic for the RSA model. The global envelope is a region of constant width 2*w_max_*, where *w_max_* is determined as the furthest deviation between *L* and any of the *L* functions of a separate set of 99 RSA simulations with the same parameters at any distance *d* along the horizontal axis. We rejected the null hypothesis if the *L* function of the sample lay outside the envelope for any value of *d* (see Section 3.2 and Figure 3).

We analyzed spatial patterns using R software and the **spatstat** package (Baddeley and Turner, 2005; Baddeley, 2010). We obtained the translation edge-correction estimator of Ripley's *K* function in three dimensions for both the observed samples and the RSA simulations using the *K3est* function included in the **spatstat** package and we directly calculated the *L* functions from *K* functions using Equation (3). To compute the simulation envelopes of the *L* functions we used the *envelope.pp3* function, also included in the **spatstat** package. We used this function with the three-dimensional point pattern for each sample and 198 three-dimensional point patterns of RSA simulations performed for that sample.

#### 2.2.3. Replicated spatial point patterns

Replicated spatial point patterns are a particular situation in the spatial point processes field where different patterns are considered as instances of the same process and are said to form a group. In our case we have several samples of each layer of the somatosensory cortex, so we conducted an analysis in the context of replicated patterns.

Let *n_ij_* (*j* = 1, …, *m_i_*) be the number of synapses for the *j*th sample in the *i*th group (*i* = 1, …, *g*). Given an estimate of the *K* function for each sample *j* in each group *i*, $\hat{K}$_*ij*_(*d*), the estimated mean function for each group is defined as

(5) $${\bar{K}}_{i}(d)=\frac{\sum_{j=1}^{m_{i}}w_{ij}{\hat{K}}_{ij}(d)}{\sum_{j=1}^{m_{i}}w_{ij}},\;i=1,\ldots ,g.$$

Different weights *w_ij_* have been proposed in the literature for function aggregation; see Pawlas (2011) for a review. Myllymäki et al. (2012) chose to use *w_ij_* = *n*^2^_*ij*_ to aggregate *K* functions together with linear mixed models to investigate the spatial structure of epidermal nerve fibers. Jafari-Mamaghani et al. (2010) used *w_ij_* = *n_ij_* to study the three-dimensional distribution of pyramidal neurons in the mouse barrel cortex. The weight *w_ij_* = *n_ij_* was also recommended by Diggle (2003). In this paper, we also chose this option.

We performed the Diggle test (Diggle et al., 1991, 2000) to study similarities and differences between groups of replicated data. This test uses a bootstrap procedure to check whether there are significant differences between empirical *K* functions of independent replicates. Using 5000 bootstrap iterations, we studied whether there were differences between the study animals and between different cortical layers.

It is scientifically correct to construct an aggregated estimator of the *K* function without assuming a common intensity across all replicates because the *K* function is defined as independent of the intensity. This assumes that the hypothesis of a common *K* function and varying intensity is plausible, as would be the case if the replicates were different intensity versions of a common underlying process (Diggle, 2013). To test if this applied in our case, we adjusted a global spatial model for groups of replicates in which the Diggle test found no significant differences. Then we applied different random thinning procedures (i.e., randomly deleting points from the original model) and introduced a cross-validation technique to honestly estimate the goodness-of-fit of the resulting models.

More explicitly, assume that *A*, *B*, and *C* were the groups where the Diggle test found no significant differences, and let *m_A_*, *m_B_*, and *m_C_* be the number of samples in each group. We adjusted the global spatial model RSA_*global*_ with parameters μ_*global*_, σ_*global*_, and λ_*global*_. Parameters μ_*global*_ and σ_*global*_ were obtained by fitting the lognormal distribution of Feret's diameters considering all synapses of all samples from groups *A*, *B*, and *C* and were used to estimate the size of the synapses in the global model. Let λ_*ij*_ be the synaptic density for the *j*th sample in the *i*th group, λ_*global*_ was chosen such that λ_*global*_ > λ_*ij*_ for all *i*,*j*, i.e., we considered a global model that was *denser* than each of the samples separately (we chose to make λ_*global*_ 1% denser than the maximum density of each sample separately).

Our goal, then, was to check whether groups *A*, *B*, and *C*, whose *K* functions were found not to be significantly different, were different thinned versions of a common underlying process. In other words, we wanted to find out whether the processes that described the spatial distribution of samples from groups *A*, *B*, and *C* were different thinned versions of the global spatial model RSA_*global*_.

**Figure 4:**
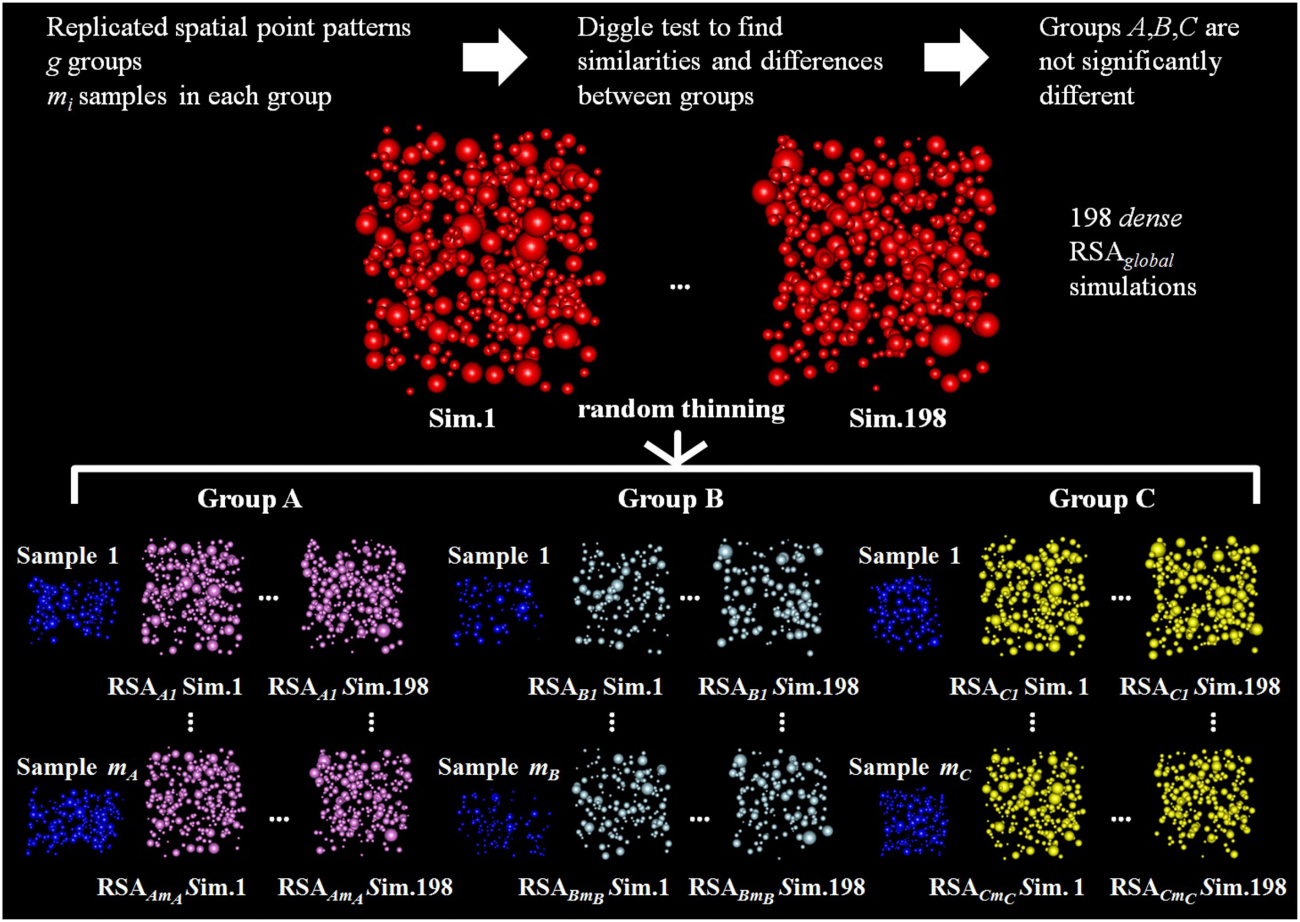
**Diagram of the random thinning process for three groups of replicated point patterns, A, B, and C, for which the Diggle test did not find significant differences**. Our goal is to check if these groups are differentially thinned versions of a common underlying RSA process. Random thinning of *dense* simulations is performed for each experimentally observed sample *j* in each group *i* (test sample, shown in blue). Random thinning continues until we reach the intensity $\hat{\lambda }$_*ij*_, estimated from all samples in group *i* excluding sample *j*. Then, for each experimentally observed sample *j* in each group *i*, we used simulation-based envelopes to test for differences in the spatial distributions of the thinned RSA simulations and the sample (we used 99 thinned simulations to estimate the *L* function for the RSA_*ij*_ model and the other 99 to calculate the maximum deviation necessary to build the envelope).

To do this, we ran 198 *dense* RSA_*global*_ simulations with the estimated parameters μ_*global*_, σ_*global*_, and λ_*global*_. Then we thinned each of these *dense* simulations for each sample in each group. We used a cross-validation technique to check if these simulations had the same spatial distribution as the experimentally observed sample. Specifically, we applied the following cross-validation process for each sample *j* (test sample) in each group *i*:

1. First, we estimated $\hat{\lambda }$_*ij*_ using the remaining (*m_i_* − 1) samples (training samples) in group *i*. The aggregated $\hat{\lambda }$_*ij*_ was calculated by weighting the densities of the training samples by their volume as in Equation (4).
2. Second, we randomly thinned the 198 *dense* RSA_*global*_ simulations until we obtained an intensity equal to the estimated density $\hat{\lambda }$_*ij*_. Thus we obtained a set of 198 thinned RSA_*ij*_ simulations for sample *j* of group *i*. These simulations were like the original simulations but had a density equal to the intensity estimation for the test sample. This process is shown in Figure 4.
3. Finally, we again used simulation-based envelopes to test for differences in the spatial distributions of the thinned simulations and the experimentally observed sample. We used 99 simulations to estimate the theoretical mean value of the L function for the RSA_*ij*_ model. We used the other 99 to calculate the maximum absolute difference from this theoretical mean value, which is necessary to build the envelope.

## 3. Results

We obtained 25 samples from the six layers of the somatosensory cortex of three 14-day-old rats by FIB/SEM microscopy. We had a total reconstructed tissue volume of approximately 4500 μm^3^ containing almost 4000 3D reconstructions of synapses. For each of these synapses, we had information on its 3D position (center of gravity or centroid) and an estimate of its size based on Feret's diameter. We obtained the density of each sample, that is, the number of synapses per unit volume, and the mean density for each layer (Table 1).

### 3.1. Synapse density in different layers

**Figure 5:**
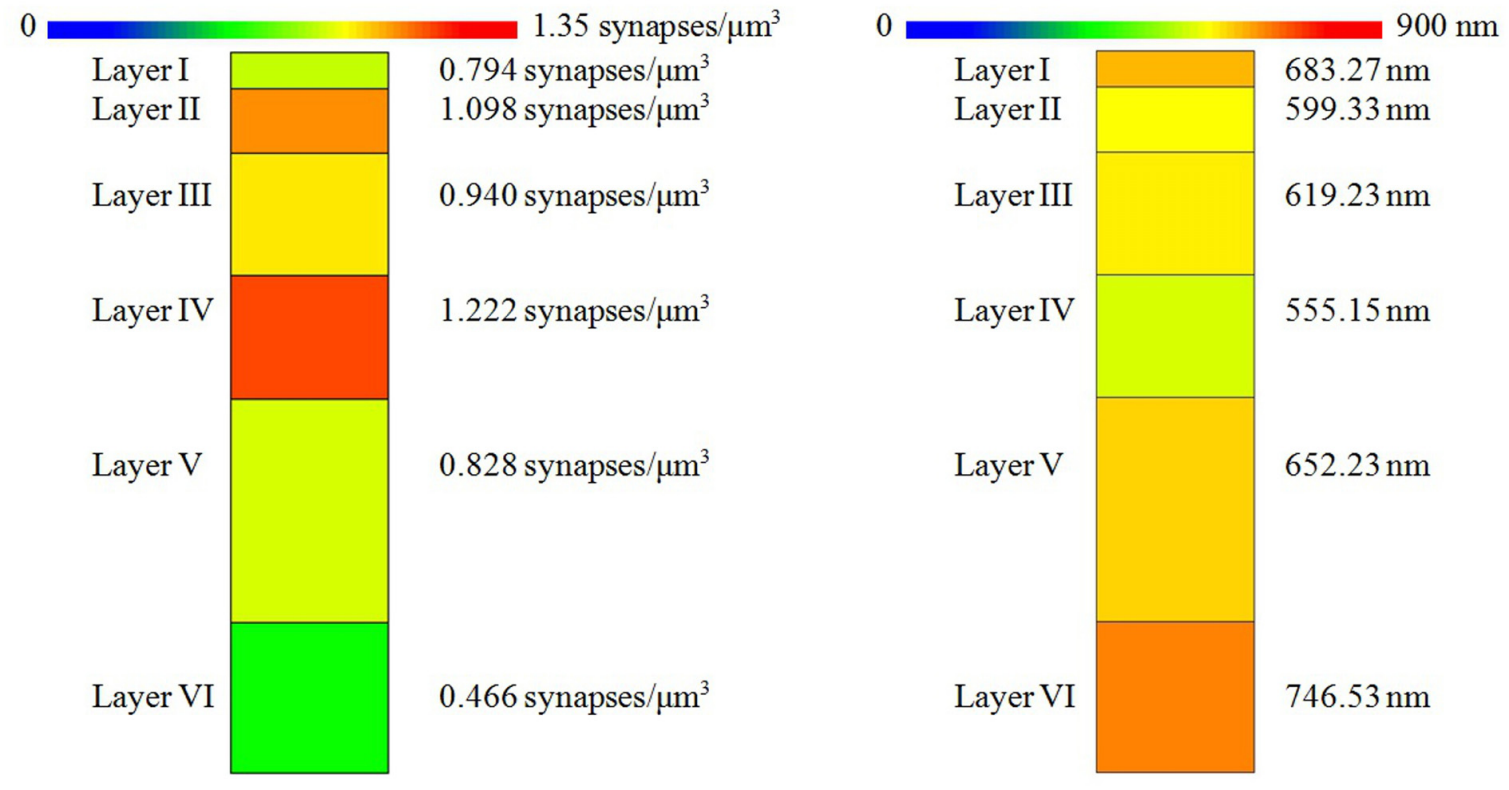
**(Left) Mean synaptic density of the six layers of the somatosensory cortex**. The synaptic density of the six layers is significantly different. However, we found no significant differences between the densities of layers I vs. V or between the densities of layers II vs. III. **(Right)** Mean distance to nearest synapse for each layer. Nearest synapse distances are significantly different in the six layers of the somatosensory cortex, but we found no significant differences between distances of layers I vs. V, I vs. VI, II vs. III, and III vs. V.

The density of the samples range from 0.382 synapses/μm^3^ in a sample of layer VI to 1.382 synapses/μm^3^ in a sample of layer II. The overall mean density is 0.870 synapses/μm^3^ in all layers. See Table 1 for details. As shown in Figure 5, the mean density of layer I is 0.794 synapses/μm^3^, whereas layers II and III have mean densities of 1.098 and 0.940 synapses/μm^3^ respectively, which increases up to the maximum mean density of 1.222 synapses/μm^3^ in layer IV and then drops again in layer V (0.828 synapses/μm^3^) down to the minimum mean density in layer VI, 0.466 synapses/μm^3^.

Following the simulation and mean comparison process described in Section 2.2.1, we looked for significant differences between the densities of the different layers of the somatosensory cortex. Using the Kruskal-Wallis test we found that there were differences between the density of layers (*p*-value ≤ 2.2 × 10^−16^), which is consistent with a recent work (Crandall, 2013). Pair-wise comparisons revealed that there was no significant difference between the densities of layers I vs. V or between the densities of layers II vs. III.

Apart from density analysis, one of the first steps often performed to explore the spatial distribution of a spatial pattern is to obtain the distance to the nearest neighbor. So, in addition to the location and Feret's diameters of synapses of each sample, which were on average 404.73 nm, we measured the distance of each synapse to its nearest synapse. The mean distances to nearest neighbor measured between centroids of synaptic junctions ranged from 533.78 nm in a sample of layer II to 794.63 nm in a sample of layer VI, and the overall mean distance to the nearest synapse was 641.58 nm. This information is shown in Table 2. Using the Kruskal-Wallis test we found that there were significant differences between the distances to the nearest synapse between layers of the somatosensory cortex (*p*-value ≤ 2.2 × 10^−16^). We applied the Mann-Whitney test and adjusted the *p*-values using the Bonferroni method for pair-wise comparisons. There were no significant differences for layers I vs. V, I vs. VI, II vs. III, and III vs. V. Notice that we found no differences between the synaptic densities of layers I vs. V and II vs. III either (see Figure 5).

**Table 2:** **Mean distances from a synapse to its nearest neighbor and mean Feret's diameters**.

	**Sample**	**Mean distance to nearest neighbor (nm) ± *SD***	**Mean Feret's diameter of synaptic junctions (nm) ± *SD***
Layer I
	1	682.09 ± 201.96	459.01 ± 196.20
	2	684.95 ± 242.28	442.01 ± 207.62
Layer II
	1	613.06 ± 191.74	429.69 ± 183.35
	2	680.80 ± 204.30	453.67 ± 184.03
	3	533.78 ± 177.72	340.96 ± 143.25
Layer III
	1	600.10 ± 193.62	377.19 ± 159.63
	2	680.33 ± 200.79	462.18 ± 177.52
	3	620.15 ± 206.34	437.62 ± 168.04
	4	615.28 ± 208.79	414.22 ± 169.04
	5	647.70 ± 228.39	466.03 ± 215.91
	6	605.46 ± 231.85	423.38 ± 169.83
	7	599.08 ± 244.67	397.29 ± 168.22
	8	643.36 ± 193.31	427.90 ± 168.15
	9	580.30 ± 203.76	378.35 ± 166.60
	10	625.62 ± 209.32	405.43 ± 175.62
Layer IV
	1	562.38 ± 228.22	397.83 ± 155.06
	2	539.84 ± 208.77	354.90 ± 129.26
	3	564.29 ± 214.38	353.52 ± 134.01
Layer V
	1	701.03 ± 235.69	414.84 ± 161.68
	2	632.66 ± 263.23	380.71 ± 173.12
	3	641.75 ± 216.35	404.49 ± 186.79
Layer VI
	1	730.74 ± 272.02	425.60 ± 146.11
	2	766.04 ± 371.24	394.42 ± 176.28
	3	694.07 ± 301.23	325.66 ± 114.03
	4	794.63 ± 357.46	351.45 ± 153.30

*Nearest neighbor distances are measured between centroids of synaptic junctions. Feret's diameters are an estimate of the size of synaptic junctions (diameter of the smallest sphere circumscribing each junction)*.

### 3.2. Modeling of spatial point processes

A recent paper (Merchán-Pérez et al., 2014) analyzed the three-dimensional spatial distribution of synapses in the somatosensory cortex. Merchán-Pérez and colleagues adjusted CSR and RSA models showing that RSA processes modeled the synaptic distribution more adequately. However, this study was limited to layer III of the somatosensory cortex. We extend this analysis to all layers of the cortex here.

To test the null hypothesis of RSA we used simulation-based envelopes. Figure 3 shows the envelopes of the first sample of each layer of the somatosensory cortex (the envelopes for all samples are shown in the Supplementary material). The averages of the *L* functions of 99 RSA simulations performed for each sample are represented in green. The shaded area is a region of constant width 2*w_max_*. The width *w_max_* was calculated with a separate set of 99 RSA simulations as described in Section 2.2.2 using the **spatstat** package. The dashed red lines show the theoretical value for CSR for visual comparison only.

The null hypothesis is rejected if the *L* function of the experimentally observed sample (blue) lies outside the envelope for any value of distance *d*. The *L* functions of samples 2 and 7 from layer III and sample 2 from layer IV were very close to the upper boundary of the envelope at a distance of about *d* = 300 nm but did not lie outside the envelope. The remaining samples were completely within the envelope for all values of *d*. So, we did not reject the RSA model for any of the 25 analyzed samples.

### 3.3. Replicated spatial point patterns

Taking advantage of the fact that we had several samples of each layer of the somatosensory cortex, we used replicated spatial point patterns in order to detect similarities and differences between groups. Because we had seen that synaptic densities between layers of the somatosensory cortex were different, we used the *K* function because it does not depend on intensity. We aggregated the *K* functions of each group using the number of synapses, as explained in Section 2.2.3 [*w_ij_* = *n_ij_*, Equation (5)] (Diggle et al., 1991; Diggle, 2013).

**Figure 6:**
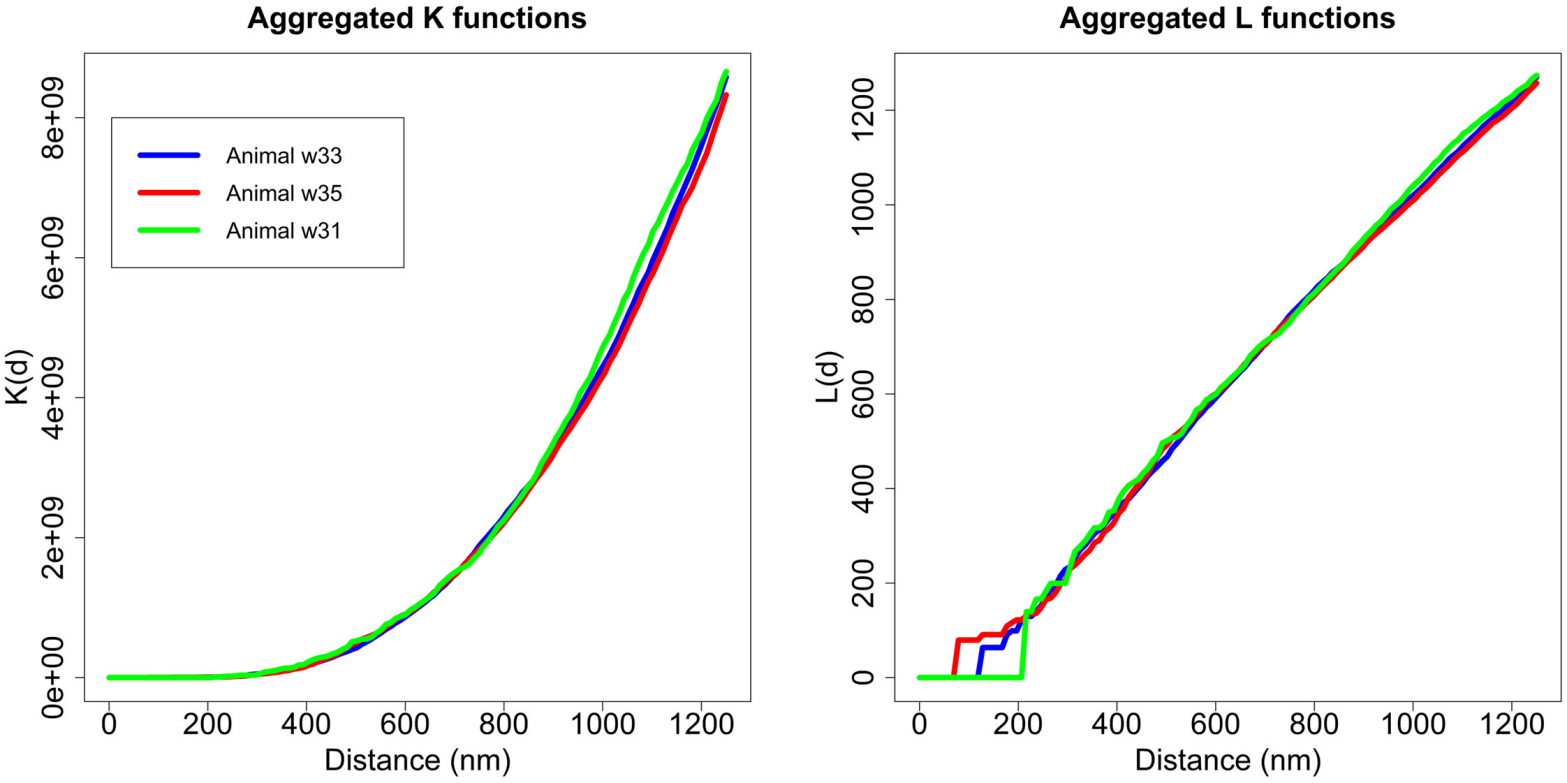
**Aggregated *K* and *L* functions for each animal**. The Diggle test found no significant differences between the three animals used in the study.

As discussed, we performed the Diggle test to compare different groups of *K* functions (Diggle et al., 1991, 2000). The first step was to check whether there were any differences between the three animals. We applied the Diggle test to *g* = 3 groups of sizes *m*_1_ = 12, *m*_2_ = 9 and *m*_3_ = 4 and obtained a *p*-value = 0.724. Thus, we did not detect differences between animals in the study. Figure 6 shows the aggregated *K* and *L* functions for each of the three animals. After ruling out differences between animals, we studied whether there were differences in the synaptic distribution between layers.

**Figure 7:**
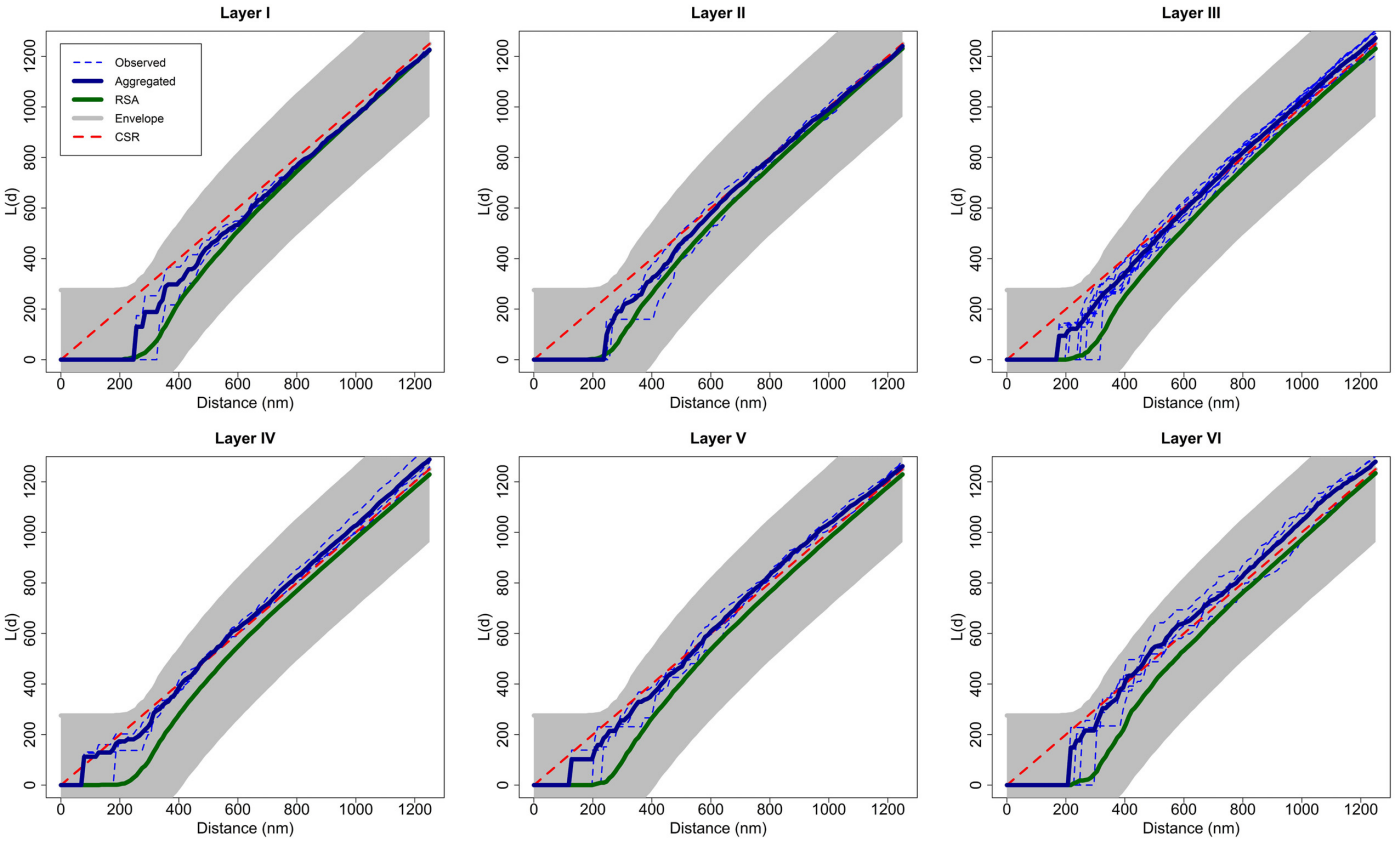
**For each layer, aggregated *L* function (dark blue) of experimentally observed data (dashed blue) along with the average of 99 RSA simulations (green) fitting the model for all samples of the layer**. This figure shows the envelope obtained using a separate set of 99 RSA simulations. We do not reject the RSA model for any layer of the somatosensory cortex because all the aggregated *L* functions were within the boundaries of the envelopes. We added the theoretical *L* function for a random pattern (dashed red diagonal) for the purpose of visual comparison.

Considering each layer of the cortex as a group of replicates, we calculated the aggregated *L* function of each group transforming the aggregated *K* function of the group [Equation (5)]. Figure 7 shows the *L* function of each observed sample in each layer as dashed blue lines, the aggregated *L* function of each layer in dark blue and the average of 99 RSA simulations fitting the RSA model for all the samples of the layer in green. We calculated the parameters $\hat{\lambda }$_*i*_, $\hat{\mu }$_*i*_, and $\hat{\sigma }$_*i*_ of the RSA_*i*_ model for each layer *i*, *i* = I,…, VI, calculating the volume-weighted average of the parameters λ_*ij*_ of each sample *j* in layer *i* and fitting the lognormal distribution of Feret's diameters using all synapses in this layer. Figure 7 also shows the envelope obtained using a separate set of 99 RSA simulations with the same parameters, as explained in Section 2.2.2. For visual comparison, we added the theoretical *L* function for a random pattern (dashed red diagonal). Because all the aggregated *L* functions were within the boundaries of the envelopes, we did not reject the RSA model for any layer of the somatosensory cortex.

**Figure 8:**
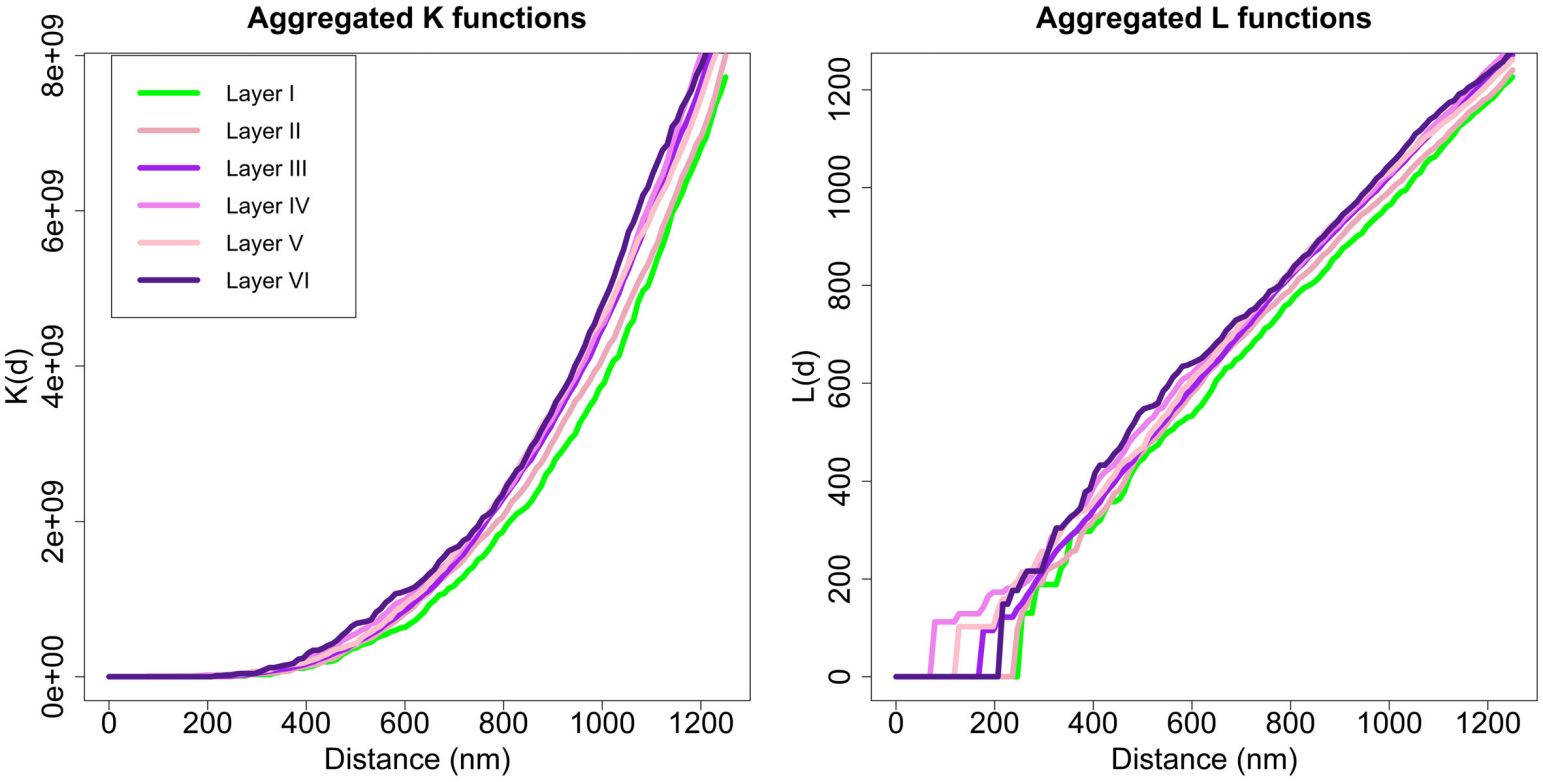
**Aggregated *K* and *L* functions for each layer**. The Diggle test found no significant differences between *K* functions of layers II, III, IV, V, and VI (shown in different shades of violet). Layer I (green) is significantly different from other layers.

Applying the Diggle test for *g* = 6 groups of sizes *m*_1_ = 2, *m*_2_ = 3, *m*_3_ = 10, *m*_4_ = 3, *m*_5_ = 3, and *m*_6_ = 4, we obtained a *p*-value of 0.002. Thus, we could conclude that there were differences between the six layers of the cortex. To better understand synaptic spatial distribution, we applied the Diggle test six times with *g* = 2 groups, each time forming a group with the *K* functions of all samples of one layer and the other group with the *K* function of all samples of the remaining layers. In this analysis, the group of samples from layer I was the only one significantly different from the other group (samples from layers II to VI) with a *p*-value of 0.009. The Diggle test found no significant differences between groups of replicates formed by layers II to VI (*g* = 5, *p*-value = 0.1176). Moreover, the Diggle test found no significant differences between the distribution of samples from layers II to VI in pair-wise comparisons of these layers. Figure 8 shows the aggregated *K* and *L* functions of all six layers (the two identified groups are shaded differently, i.e., layer I in green and layers II to VI in violet). Layer I functions are slightly shifted to the right compared to the other layers, so the repulsion in the spatial distribution of its synapses appears to be greater.

In Section 3.1 we saw that layers of the somatosensory cortex did not have a common synaptic density, so we wanted to find out whether we had different thinned versions of a common underlying process in layers from II to VI (Diggle, 2013). We did this analysis introducing for the first time in this context a cross-validation technique to honestly estimate the goodness-of-fit of the resulting models.

**Figure 9:**
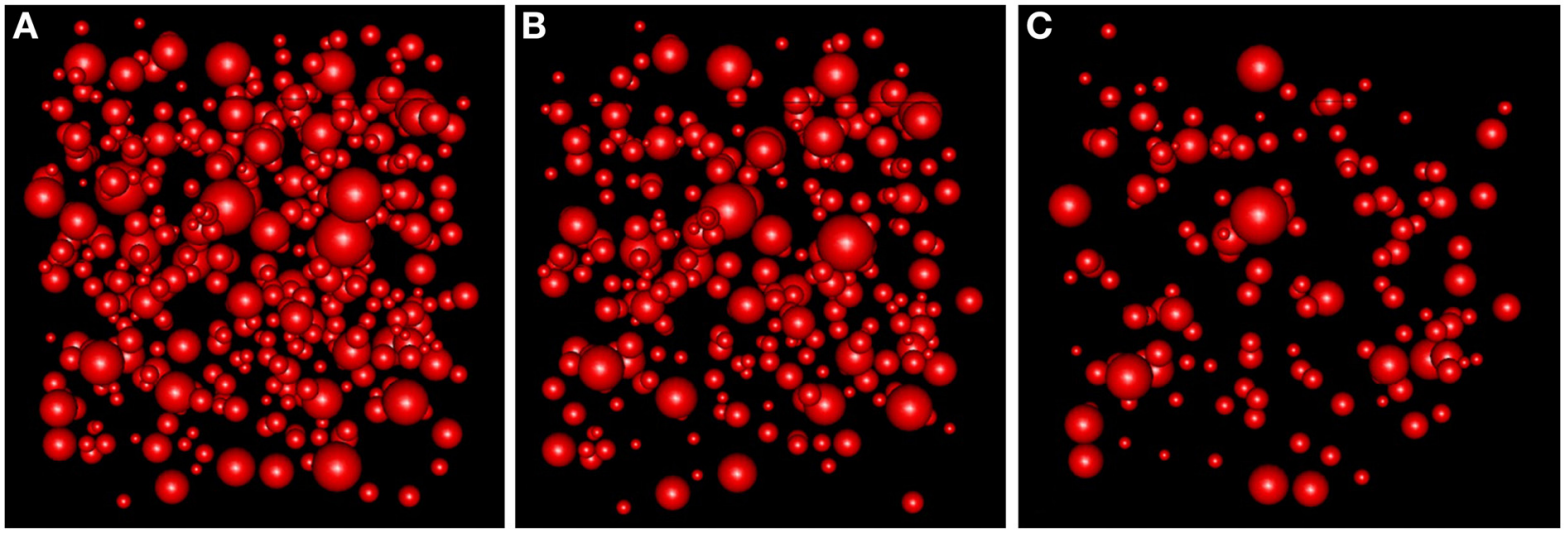
**(A)** RSA simulation with λ = 1.4 for the group of layers II, III, IV, V, and VI. **(B)** Thinned RSA simulation, λ = 0.932, for sample 10 of layer III. λ estimated from the remaining nine samples of layer III. **(C)** Thinned RSA simulation, λ = 0.457, for sample 1 of layer VI. λ estimated from the remaining three samples of layer VI.

With the simulation and thinning process described in Section 2.2.3, we performed 198 *dense* RSA_*global*_ simulations with a volume of 300 μm^3^ and a density of 1.4 synapses/μm^3^ (λ_*global*_ = 1.4, a density greater than the density of any of the samples), i.e., each RSA_*global*_ simulation had 420 synapses. For each sample *j* (test sample) in group *i* (we had a group consisting of layers II to VI), we calculated the synaptic density of its RSA_*ij*_ model using the remaining samples of the same layer [Equation (4)]. Table 3 shows the estimated intensity $\hat{\lambda }$_*ij*_ for each experimental sample. For each sample, we randomly thinned each of the 198 *dense* RSA_*global*_ simulations until they had the estimated intensity $\hat{\lambda }$_*ij*_. The sizes of the simulated synapses were calculated using the lognormal distribution fitted using Feret's diameters of all samples of the group. Table 3 also shows these parameters. Note that μ_*global*_ and σ_*global*_ are equal because all these layers were modeled as a common RSA_*global*_ process. Figure 9 shows one *dense* RSA_*global*_ simulation for the group of layers II to VI and two thinned RSA simulations for two different samples in the study.

**Table 3:** **Estimated intensity $\hat{\lambda }$_*ij*_ for samples in layer II to VI using only the remaining samples of the same layer [Equation (4)]**.

			**Density**	**Size (Feret's diameters)**	
	**Sample**	**Animal**	**$\hat{\lambda }$_*ij*_**	**μ_*global*_**	**σ_*global*_**
	1	w33	1.154		
Layer II	2	w35	1.168	5.911	0.404
	3	w35	0.981		
	1	w31	0.936		
	2	w31	0.963		
	3	w33	0.941		
	4	w33	0.932		
Layer III	5	w33	0.939	5.911	0.404
	6	w33	0.940		
	7	w33	0.934		
	8	w35	0.962		
	9	w35	0.919		
	10	w35	0.932		
	1	w33	1.286		
Layer IV	2	w35	1.200	5.911	0.404
	3	w35	1.186		
	1	w33	0.876		
Layer V	2	w33	0.782	5.911	0.404
	3	w33	0.821		
	1	w33	0.457		
Layer VI	2	w35	0.466	5.911	0.404
	3	w31	0.438		
	4	w31	0.508		

*μ_*global*_ and σ_*global*_ parameters are the same because layers II to VI form a group, and they were obtained using Feret's diameters of all samples of the group. We thinned RSA_*global*_ simulations modeled with λ_*global*_ = 1.4 and parameters μ_*global*_ and σ_*global*_ until we reached the estimated intensity $\hat{\lambda }$_*ij*_ for each sample*.

We validated the RSA_*ij*_ model with the test sample *i* using simulation-based envelopes. To do this, we used the function *envelope.pp3* included in the **spatstat** package. The *L* functions of sample 7 from layer III and sample 2 from layer IV touched the upper boundary of the envelope slightly at distances around 200–300 nm but did not lie outside the envelope. However, sample 1 from layer IV did lie just outside the envelope at distances around 300–400 nm (envelopes for all RSA_*ij*_ models are shown in Supplementary material). The remaining samples were completely within the envelope. Thus, for all 23 samples in layers II to VI, except for only sample 1 in layer IV, we did not reject the null hypothesis of RSA, i.e., we validated the hypothesis that the synaptic distribution of layers II to VI of the somatosensory cortex are different thinned versions of a common underlying RSA process.

## 4. Discussion

Historically, spatial point processes have been more related to applications in which data collection tended to be costly (e.g., forestry). For this reason, the study of several independent samples as realizations of the same process was not usually considered. Recently, the field of replicated point patterns is growing strongly since technological advances have simplified sampling, particularly 3D sampling. In fact, much of the research on replicated point patterns is related to biological issues, including applications to neuroanatomical data (Diggle et al., 1991, 2000; Baddeley et al., 1993; Wager et al., 2004; Jafari-Mamaghani et al., 2010; Burguet et al., 2011; Myllymäki et al., 2012; Burguet and Andrey, 2014). Indeed, neuroanatomical data in the form of spatial point patterns is fundamental for revealing the spatial architecture of the different brain regions at all levels of analysis, from light microscopy (e.g., spatial distribution of neurons) to electron microscopy (e.g., spatial distribution of synapses). In this paper, we performed an analysis in the context of replicated point patterns by exploiting the fact that we have been able to obtain a relatively large number of samples containing the spatial distribution of synapses in the neuropil from several layers of the rat cerebral cortex. Using the Diggle test (Diggle et al., 1991, 2000) we detected groups of replicates (groups of patterns considered as instances of the same process) whose spatial distribution was found not to be significantly different. Then we modeled these groups using a global RSA replicated spatial point process. In order to collect and explain the variability in each group's synaptic density, we introduced a thinning procedure in the global model. To honestly estimate the goodness-of-fit of the resulting models, we used for the first time in this context a cross-validation technique for models within each group of replicates.

Our results confirm the assumption that the spatial distribution of synaptic junctions in the neuropil is nearly random, with the only constraint that synapses cannot overlap in space—a scenario that can be modeled by an RSA process. This model had already been suggested for layer III synapses (Merchán-Pérez et al., 2014) and is now extended to all neocortical layers. We found that the spatial distribution of synapses in all samples of each layer can be described by RSA processes. We also found that the spatial distribution of synapses in the neuropil of layers II to VI follows a common underlying RSA process with different synaptic densities. Interestingly, the results showed that the synaptic spatial distribution in layer I is slightly different than in other layers, suggesting that, although an RSA process suitably fits layer I synaptic distribution, the repulsion in the spatial distribution of synapses in this layer is slightly higher than in the other layers.

Since the synaptic density in the cerebral cortex changes with age, e.g., Rakic et al. (1986, 1994); Bourgeois and Rakic (1993); DeFelipe et al. (1997), and we used P-14 rats, the conclusion of this study regarding spatial distribution may not be applicable at other time points during development. Note, however, that the spatial distribution of synapses follows the same pattern in different cortical layers in spite of significant differences in their synaptic densities. Furthermore, our preliminary results in the adult human cerebral cortex also suggest that the spatial distribution of synapses is nearly random (Blazquez-Llorca et al., 2013). Therefore, random spatial distribution of synapses is probably a common general pattern of cortical synaptic organization. Nevertheless, further studies in other cortical areas, species and ages would be necessary to verify these conclusions.

The assumption that the distribution of synapses in the neuropil of layers I to VI follows an RSA model with different intensities (synaptic densities) per layer has several interesting implications. First, the position of a given synapse in the neuropil is practically independent of the position of neighboring synapses, so they can be arbitrarily close to one another with the only physical constraint that they cannot overlap. Second, the density of synapses varies by layers and also locally. Importantly, early studies of the cerebral cortex proposed that the density of synapses was relatively constant throughout the cortical layers, as well as across different cortical areas and different species. This uniformity in synaptic density led O'Kusky and Colonnier (1982) to propose that it probably reflects the optimal number of synapses and that it may be due to some limiting metabolic or structural factor. However, most comparisons were only qualitative and not based on statistical analyses. It now appears that, using appropriate stereological counting methods (disector or size-frequency methods; see DeFelipe et al., 1999), there are significant differences in the estimated number of synapses per volume between certain layers in several species (reviewed in DeFelipe et al., 2002a). In this study, we also found using FIB/SEM that there may be significant differences between certain cortical layers. This method has the advantage that it provides the actual number of synapses per volume instead of estimations based on the analysis of single electron microscope images (Merchán-Pérez et al., 2009).

Our results showed no significant differences in the synaptic distribution between the different rats used in the study, and RSA processes properly described the spatial distribution of synapses in all cortical layers. This argues in favor of a common general principle of synaptic organization. However, the mean density of synapses across the six layers was significantly different, with the exception of layers I vs. V and layers II vs. III. This is an important observation in terms of connectivity, as these differences or similarities in density of synapses between layers may provide us with some fundamental rules to generate hypothetical circuits in order to gain a better understanding of cortical organization. This also means that, due to physical constraints, the volume of the neuropil that the dendritic tree of a given neuron occupies may vary depending of the density of neurons in the layer where this neuron is located. In turn, its chances of establishing synapses would be greater the more neuropil volume it occupies. This idea was put forward by Von Economo (1926) in his interpretation of Nissl's observation in terms of the evolutionary significance of the differences between species in cortical neuronal density (Nissl, 1898). Nissl observed that “in the mole and dog, cortical neurons were more crowded than in man.” Von Economo proposed that the greater separation between neurons the richer the fiber plexus between them will be, increasing the chance for neuronal interactions. Thus, the larger separation of neurons in humans compared to other species could be construed as a sign of a greater complexity of the connections between neurons. Using this approach, several authors have identified an inverse relationship in the adult cerebral cortex between neuronal density and the number of synapses per neuron in different cortical areas/layers/species, but this principle does not appear to be generally applicable (DeFelipe et al., 2002a). Since in this study we found no significant differences in the density of synapses in layer I vs. V—the density of neurons in layer V is much greater than in layer I—, or between layer II vs. III—the density of neurons in layer III is much less than in layer II (work in preparation)—, this principle does not appear to be applicable to the 14-days-old rat somatosensory cortex either. In this regard it is important to keep in mind that the dendrites present in the neuropil of a given layer belong to both local neurons and neurons located below and above that layer, as dendrites, of pyramidal cells particularly, may cross several layers during their ascending course toward layer I, whereas their basal dendrites may invade the layer underneath, respectively. It follows that the number of synapses that a given neuron receives cannot be predicted solely on the basis of the synaptic density of the layer in which it is located.

Finally, the application of FIB/SEM to analyze the neuropil also revealed the existence of local variability in the synaptic density within each layer. This local variability would be the product of mere chance and can be explained (and modeled) by RSA processes. The between-layers variability, however, cannot be put down to chance, except possibly for the differences between layers I and V and between layers II and III. This would imply, as previously suggested (Merchán-Pérez et al., 2014), that spatial specificity in the neocortex is scale dependent. It is well known that at the macroscopic and mesoscopic scales the mammalian nervous system is a highly ordered and stereotyped structure, where connections are established in a highly specific and ordered way, like, for example, the connecting pathways of the visual system. Even at the microscopic level, it is clear that different areas and layers of the cortex receive specific inputs (Nieuwenhuys, 1994). At the ultrastructural level, however, our results seem to indicate that number and distribution of synapses follow a nearly random pattern. This could mean that, as the axon terminals reach their destination, the spatial resolution that they achieve is fine enough to find a specific cortical layer but not to make a synapse on a smaller target, such as a specific dendritic branch or dendritic spine within that layer. For example, axon terminals from a certain thalamic nucleus reach specific areas and layers of the cerebral cortex but, once there, they would form synapses randomly among their possible targets to a greater or lesser extent depending on particular classes of the postsynaptic neurons. For instance, studies by White and colleagues performed on the mouse somatosensory cortex found a specificity of synaptic connections by combining anterograde degeneration of thalamic axonal fibers with the retrograde transport of horseradish peroxidase to identify the projection sites of pyramidal cells (revised in White, 1989). They examined at the electron microscope level pyramidal cells projecting to ipsilateral cortical areas, to the thalamus and to the striatum and they found that each of these populations of pyramidal cells receives a characteristic proportion of their layer IV dendritic synapses from thalamocortical axon terminals. Corticothalamic cells receive the greatest number of thalamocortical synapses, corticocortical cells the next highest number, and corticostriatal cells the least. Therefore, at the synaptic scale, the specificity of connections would rely not on spatial cues but on other mechanisms such as molecular or activity-dependent cues.

### Conflict of interest statement

The authors declare that the research was conducted in the absence of any commercial or financial relationships that could be construed as a potential conflict of interest.
