# Supplementary material for: Three-dimensional distribution of cortical synapses: a replicated point pattern-based analysis
Source: Front Neuroanat. 2014 Aug 26;8:85. doi: 10.3389/fnana.2014.00085 (PMC4143965; doi:10.3389/fnana.2014.00085)
Supplement: Supplementary file 1 [file DataSheet1.PDF]

Frontiers in Neuroanatomy

Research Article
14 July 2014

Supplementary Material: Three-dimensional distribution of cortical synapses: a replicated point pattern-based analysis

Laura Anton-Sanchez [1,*], Concha Bielza [1], Angel Merchán-Pérez [2,3], José-Rodrigo Rodríguez,[2,4], Javier DeFelipe [2,4] and Pedro Larrañaga [1]

[1] *Departamento de Inteligencia Artificial, Escuela Técnica Superior de Ingenieros Informáticos, Universidad Politécnica de Madrid, Madrid, Spain*
[2] *Laboratorio Cajal de Circuitos Corticales, Centro de Tecnología Biomédica, Universidad Politécnica de Madrid, Madrid, Spain*
[3] *Departamento de Arquitectura y Tecnología de Sistemas Informáticos, Escuela Técnica Superior de Ingenieros Informáticos, Universidad Politécnica de Madrid, Madrid, Spain*
[4] *Instituto Cajal, Consejo Superior de Investigaciones Científicas, Madrid, Spain*

Correspondence*:
Laura Anton-Sanchez
Departamento de Inteligencia Artificial
Escuela Técnica Superior de Ingenieros Informáticos
Universidad Politécnica de Madrid
Campus de Montegancedo s/n
Boadilla del Monte 28660 Madrid, Spain,
l.anton-sanchez@upm.es

Quantitative analysis of neuroanatomy

1 SUPPLEMENTARY FIGURES

1.1 RSA ENVELOPES

Supplementary Figures 1 to 8. Analysis of spatial patterns using global envelopes for each of the samples in layers I to VI of the somatosensory cortex. The L functions of the experimentally observed samples are shown in blue. The averages of 99 RSA simulations performed for each sample are shown in green. The shaded area represents the envelopes of values calculated from a separate set of 99 RSA simulations. Dashed red lines show the theoretical value for CSR and were added for the purpose of visual comparison. The L functions of samples 2 and 7 from layer III and sample 2 from layer IV are very close to the upper boundary of the envelope at a distance of about d =300 nm but do not lie outside the envelope. The remaining samples are completely within the envelope for all values of d. So, we do not reject the null hypothesis of RSA for any of the 25 analyzed samples.

1.2 THINNED RSA ENVELOPES

Supplementary Figures 9 to 15. Analysis of spatial patterns using global envelopes for each of the samples in the group of layers II to VI. Figures show the L functions of the experimentally observed samples (blue) and the averages of 99 thinned RSA simulations performed for each sample (green). We

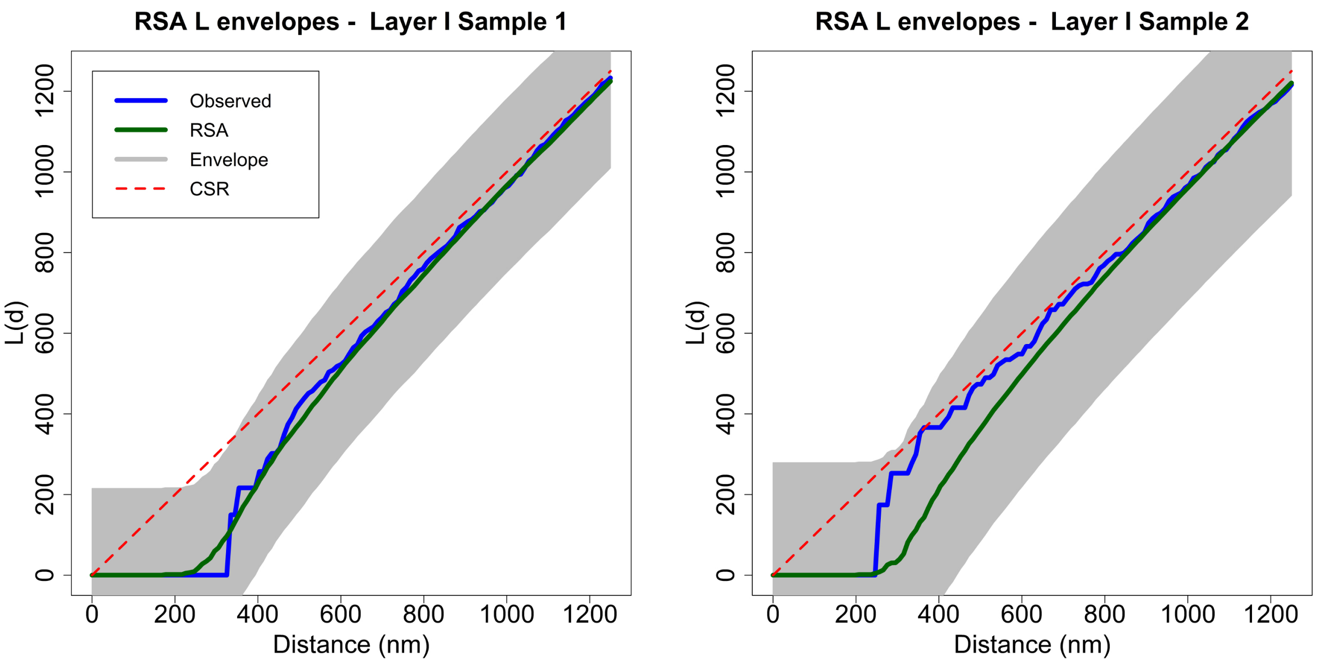

Supplementary Figure 1. Samples of Layer I

calculated the envelopes from a separate set of 99 thinned RSA simulations with the same parameters. Dashed red lines show the theoretical value for CSR (for visual comparison only). The L functions of sample 7 from layer III and sample 2 from layer IV touch the upper boundary of the envelope slightly at distances around 200-300 nm but do not lie outside the envelope. However, sample 1 from layer IV lies just outside the envelope at distances around 300-400 nm. The remaining samples are within the envelope. Thus, for all 23 samples in layers II to VI, except for only sample 1 in layer IV, we do not reject the null hypothesis of RSA, i.e., we validated the hypothesis that the synaptic distribution of layers II to VI of the somatosensory cortex are different thinned versions of a common underlying RSA process.

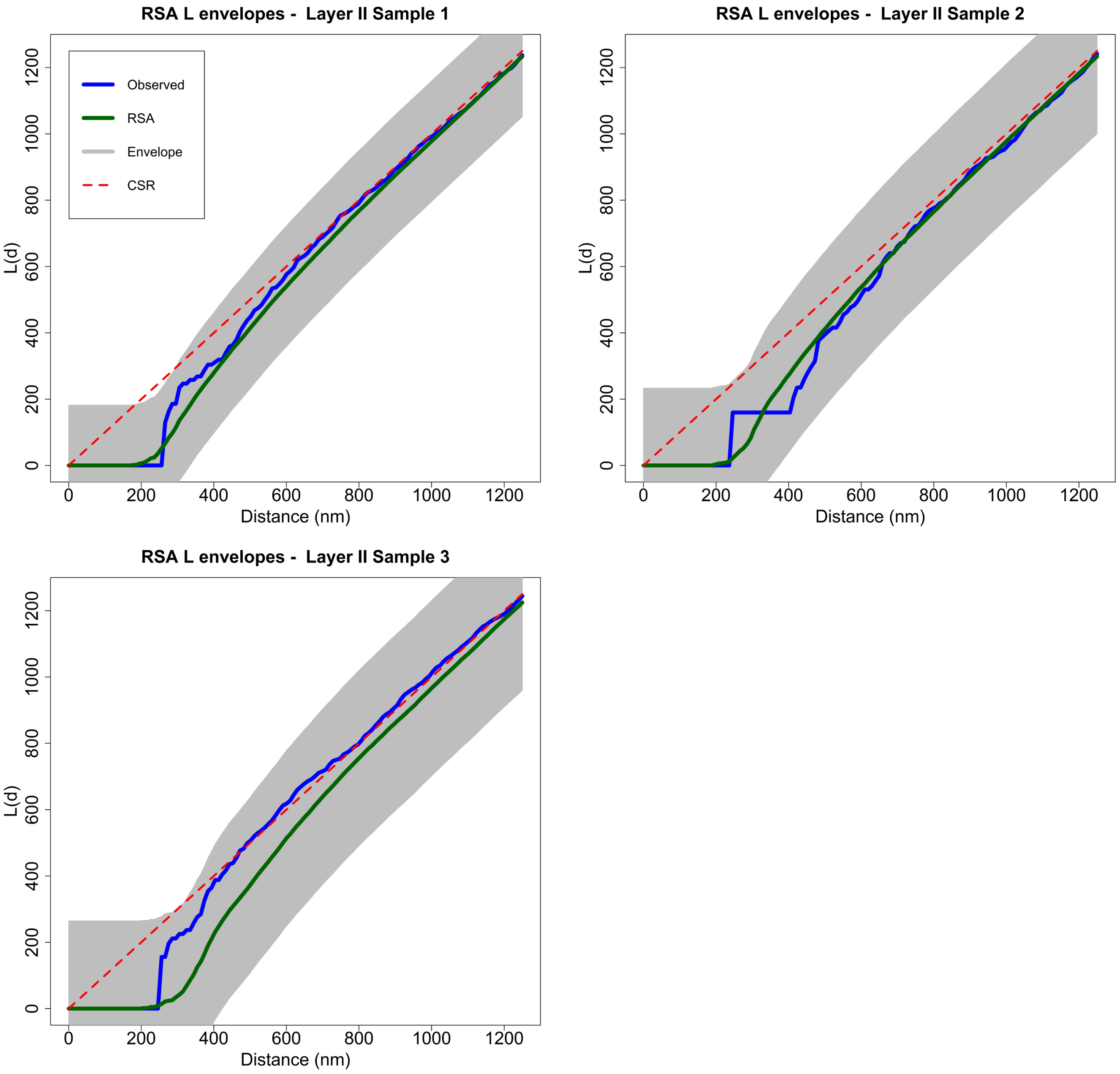

Supplementary Figure 2. Samples of Layer II

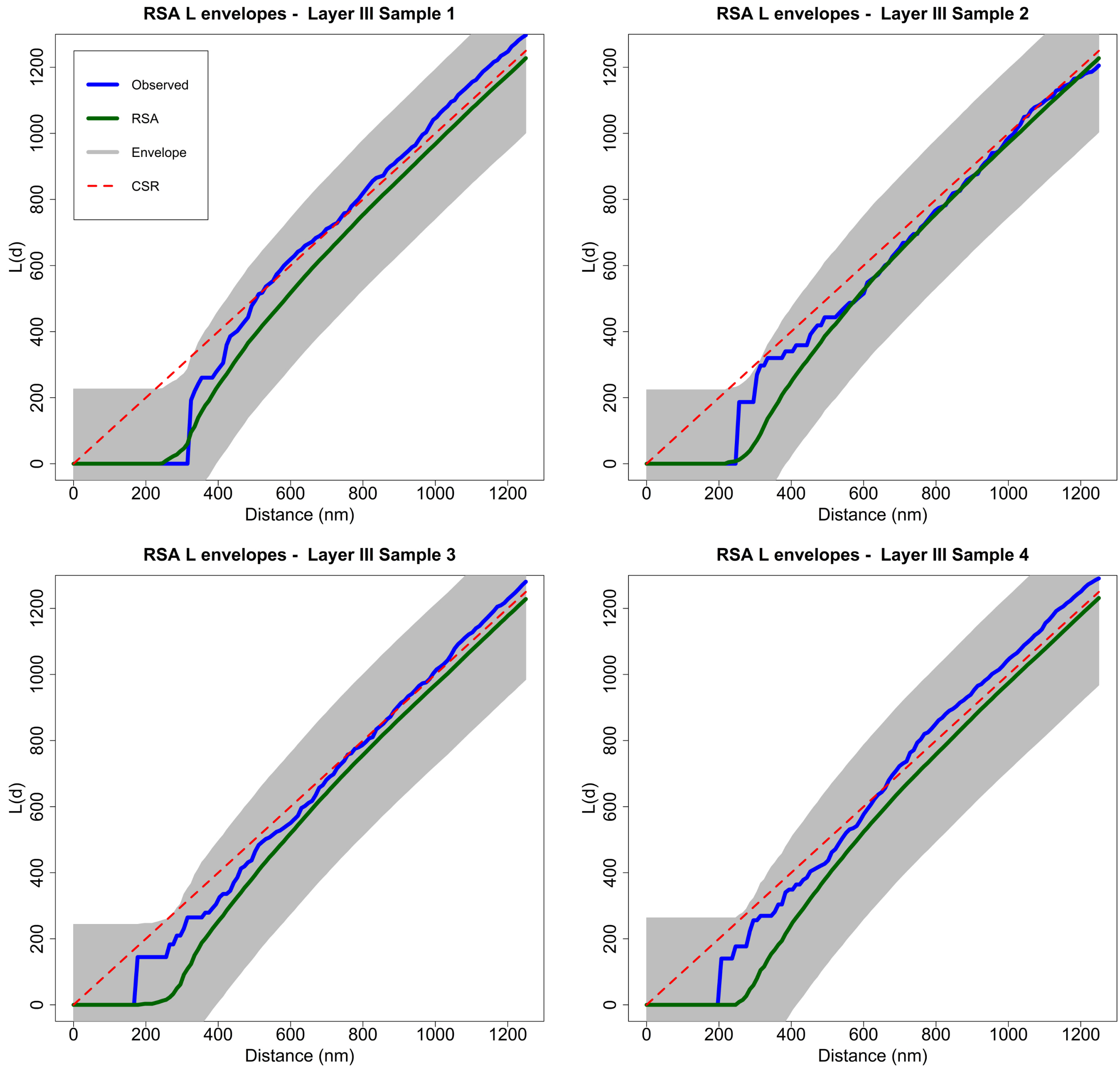

Supplementary Figure 3. Samples 1, 2, 3 and 4 of Layer IIII

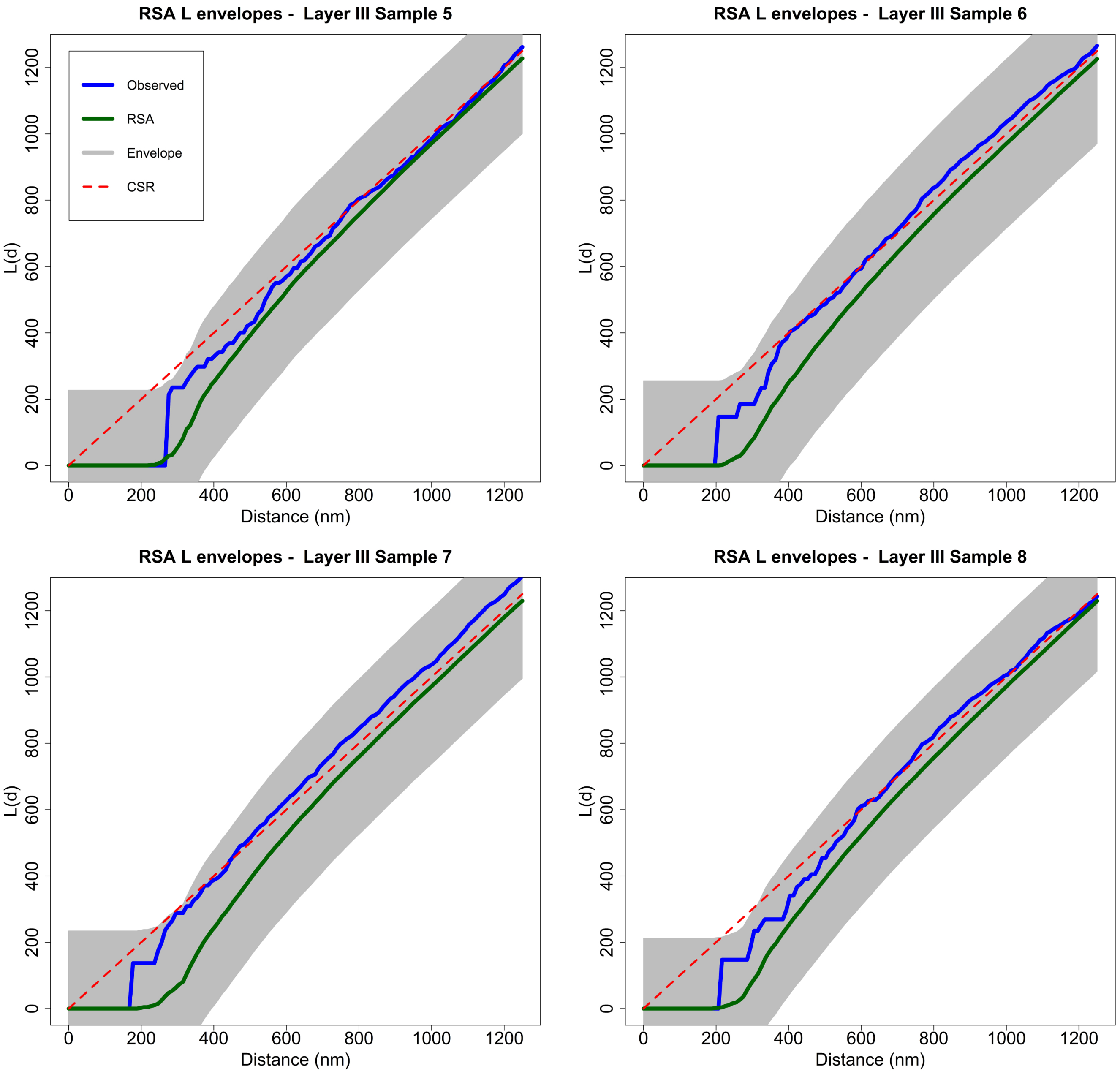

Supplementary Figure 4. Samples 5, 6, 7 and 8 of Layer IIII

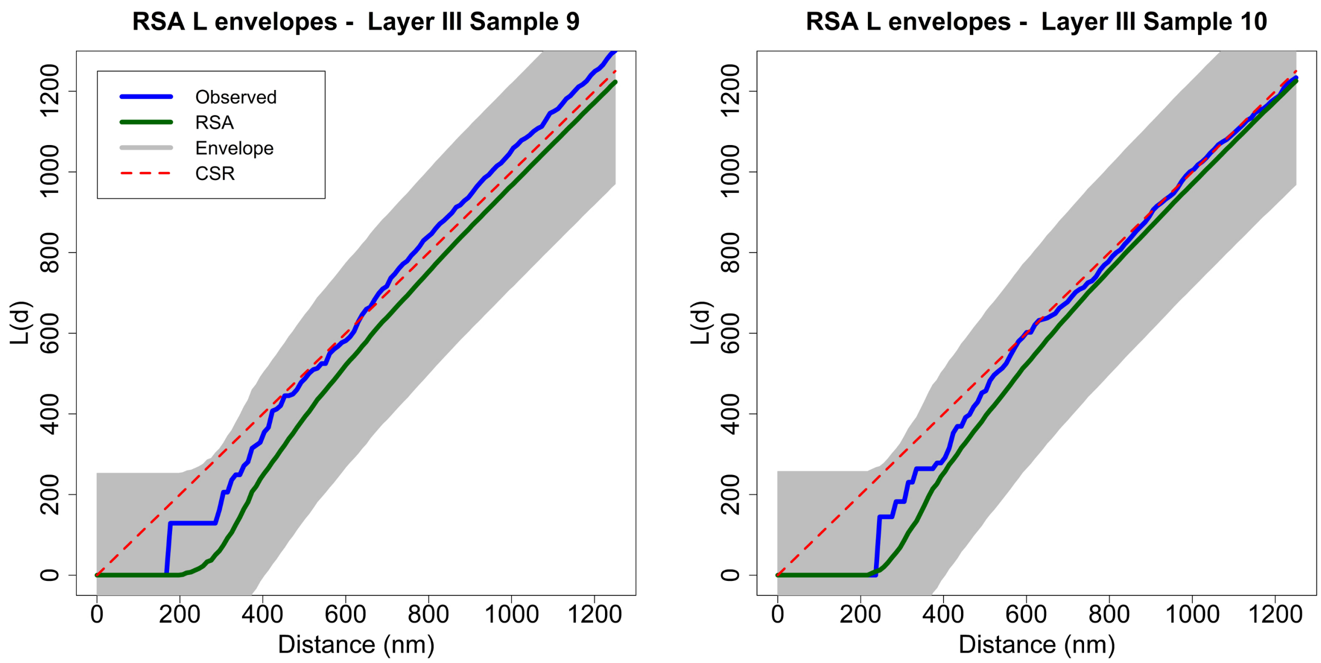

Supplementary Figure 5. Samples 9 and 10 of Layer IIII

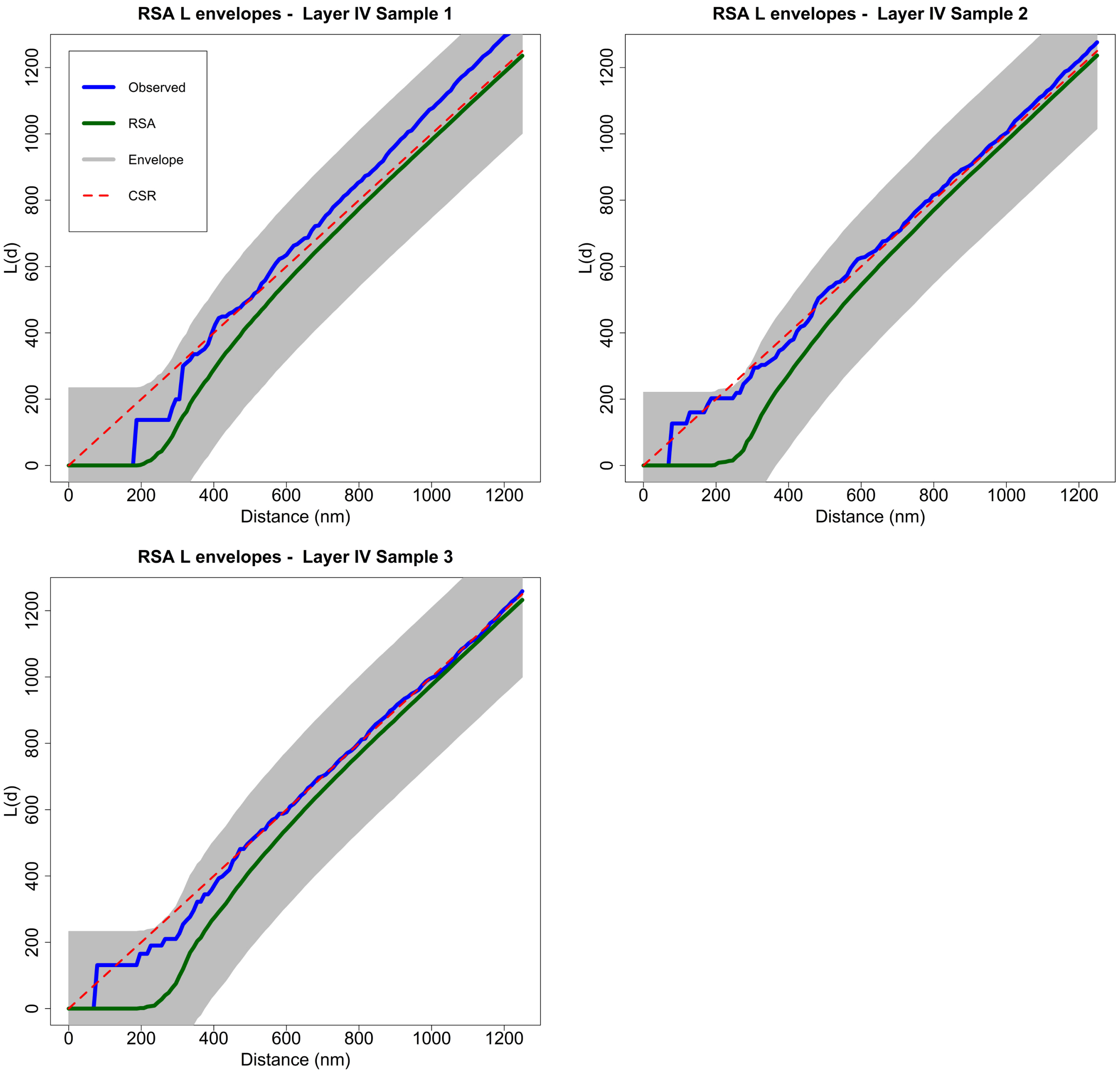

Supplementary Figure 6. Samples of Layer IV

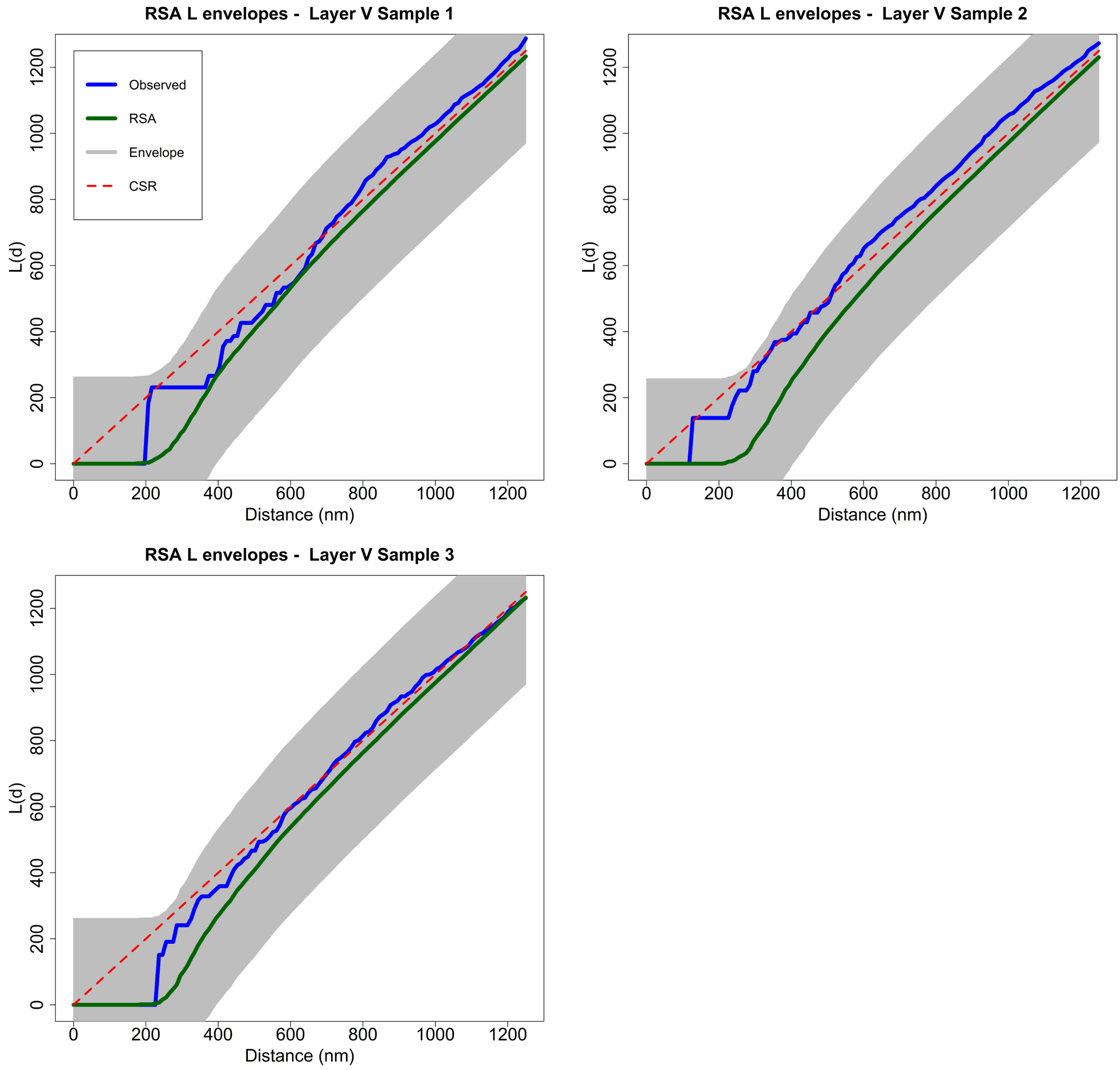

Supplementary Figure 7. Samples of Layer V

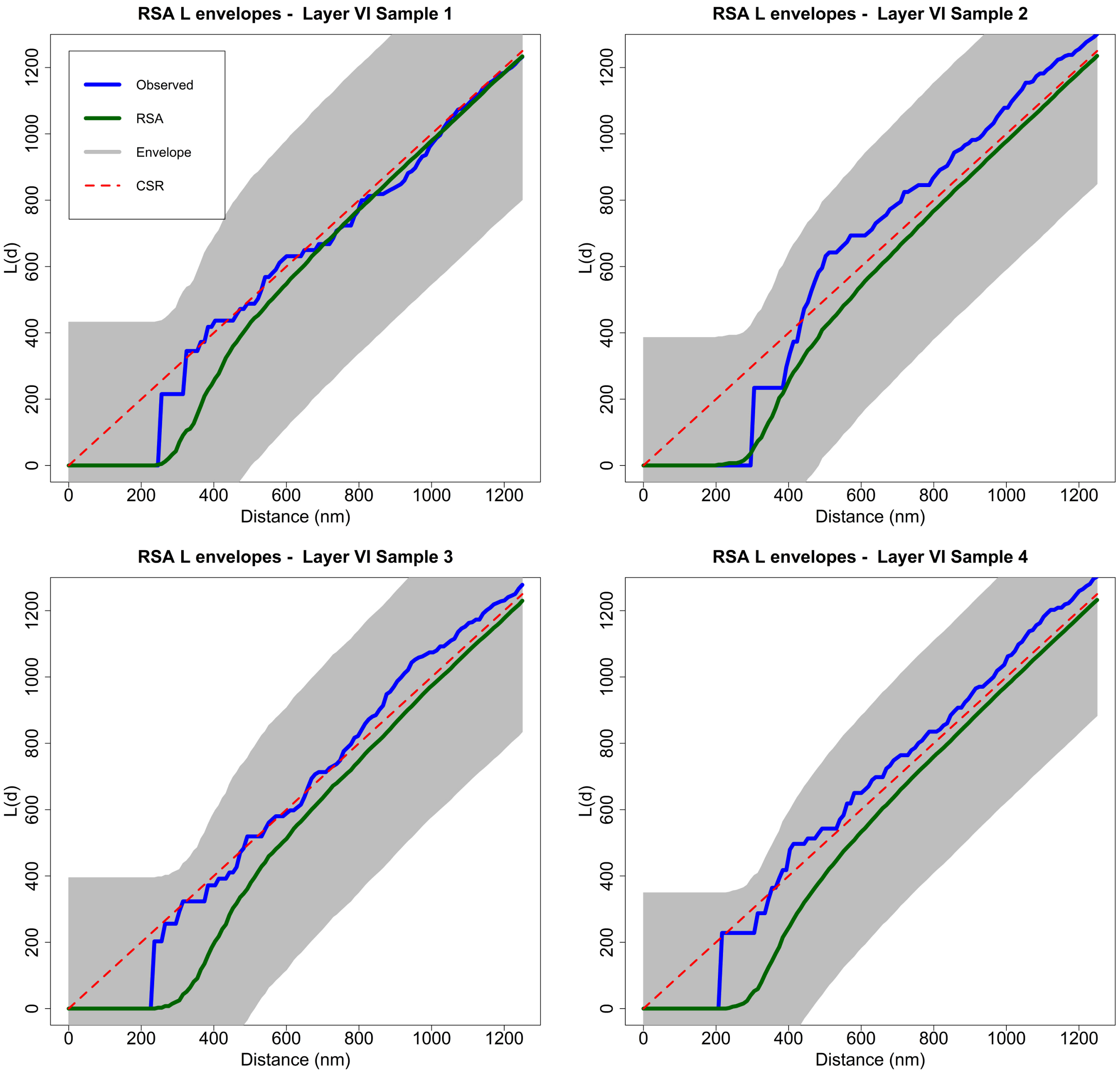

Supplementary Figure 8. Samples of Layer VI

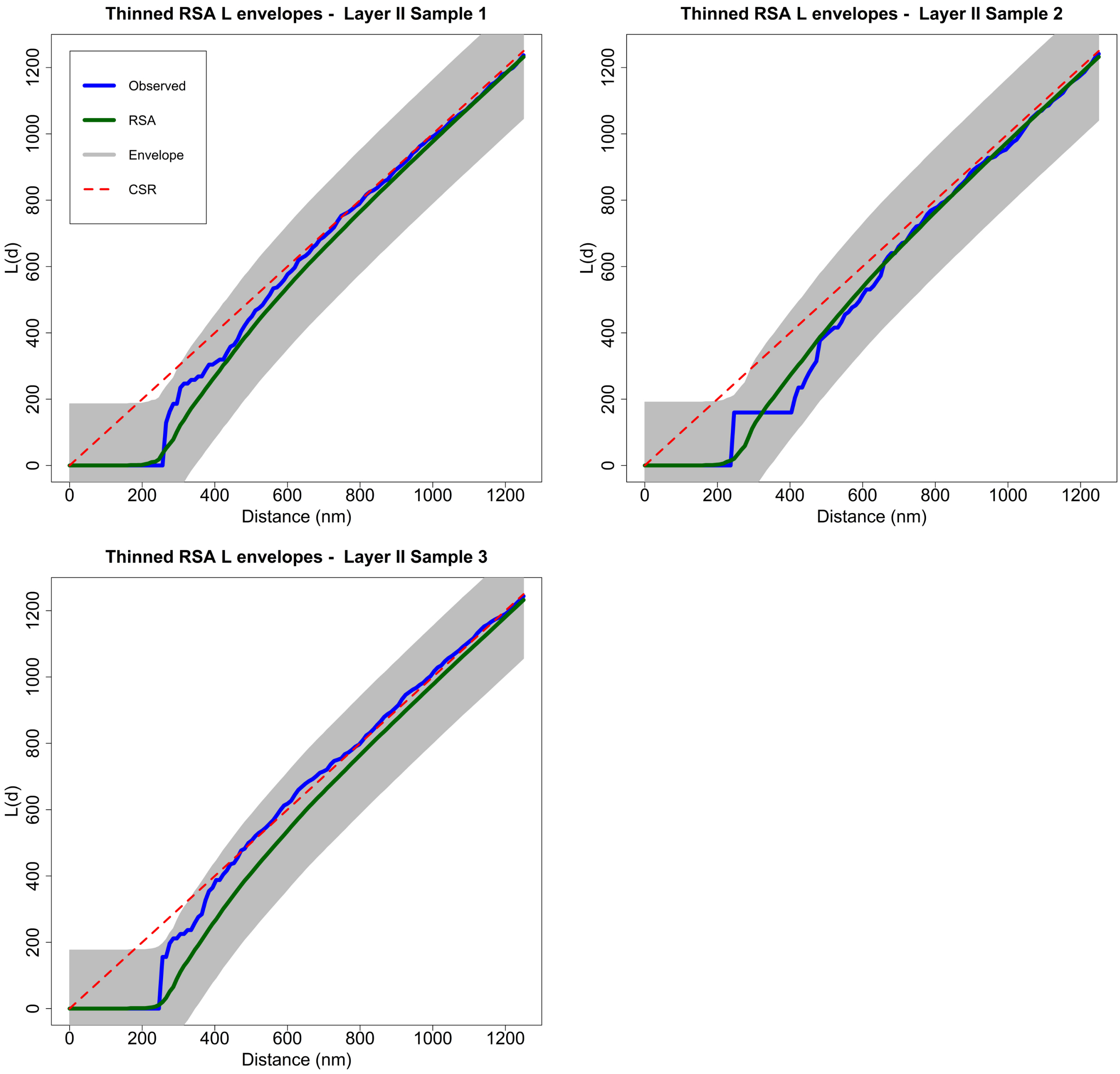

Supplementary Figure 9. Samples of Layer II

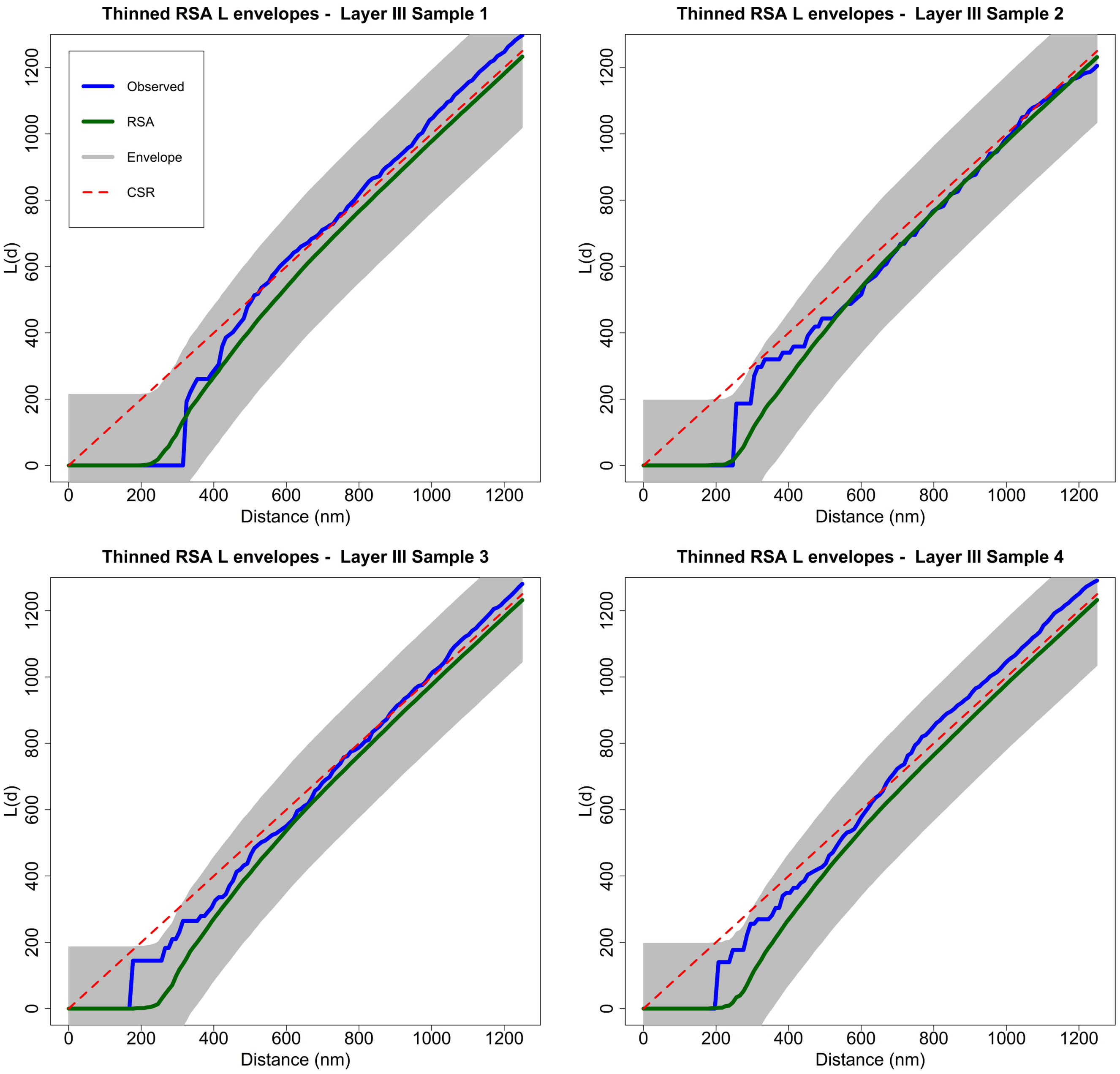

Supplementary Figure 10. Samples 1, 2, 3, and 4 of Layer III

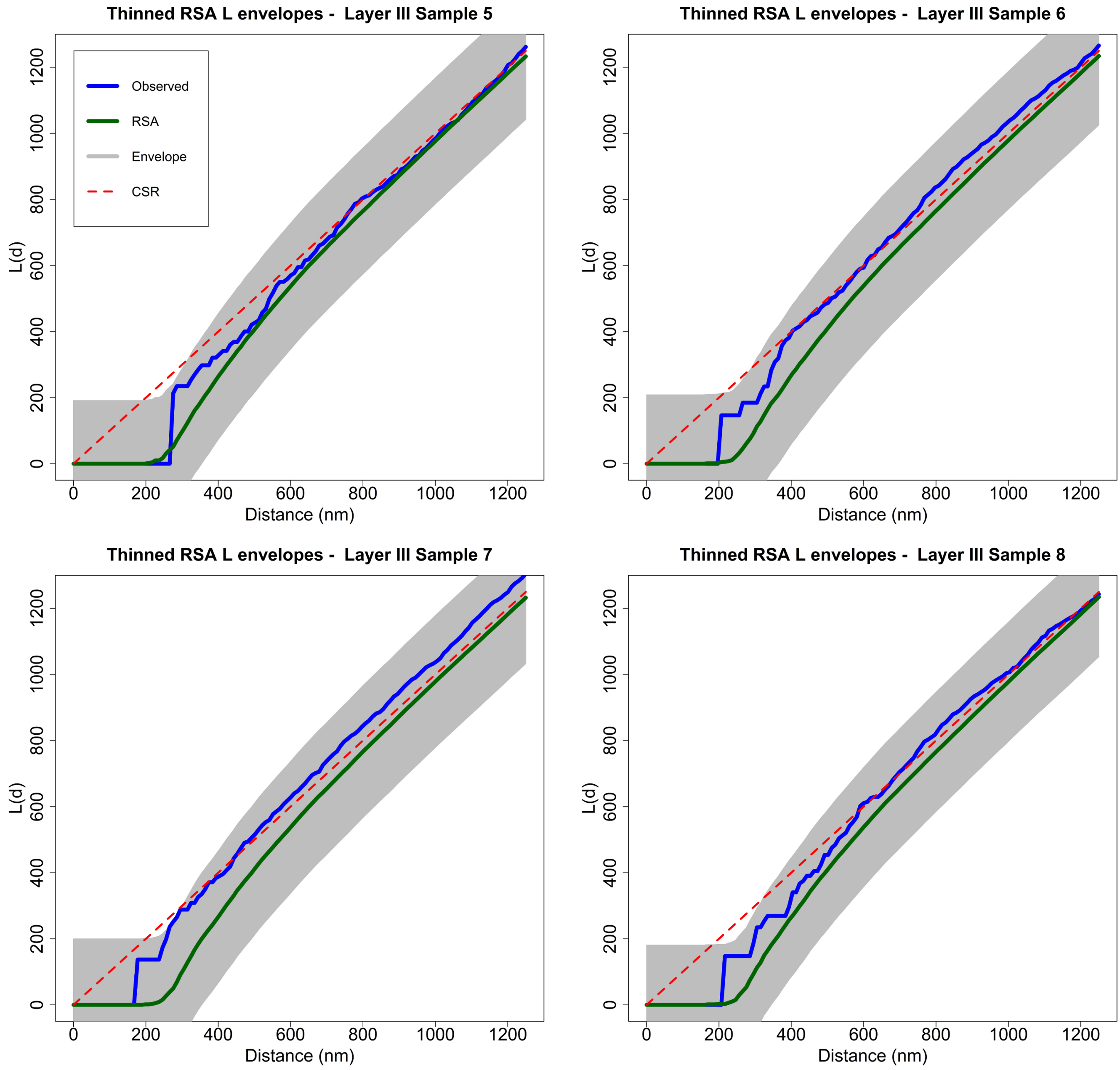

Supplementary Figure 11. Samples 5, 6, 7 and 8 of Layer III

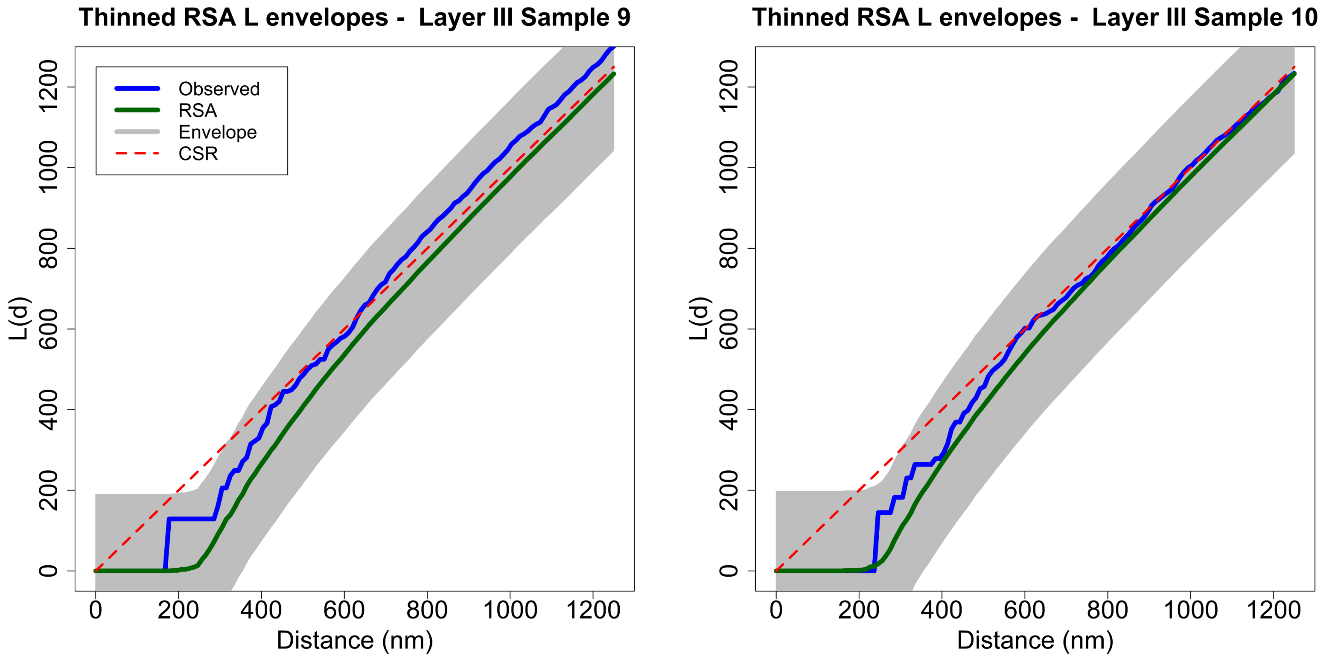

Supplementary Figure 12. Samples 9 and 10 of Layer III

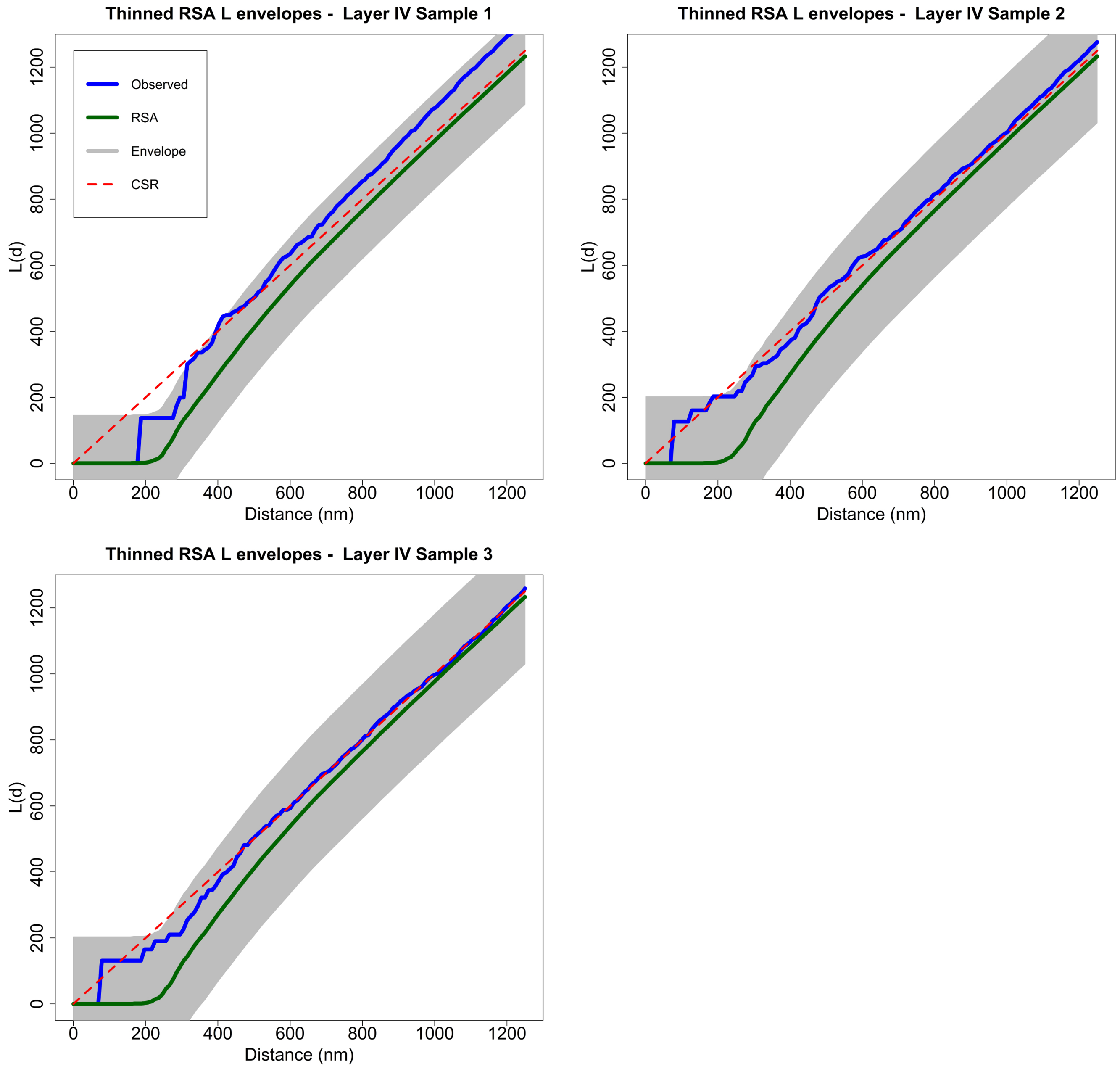

Supplementary Figure 13. Samples of Layer IV

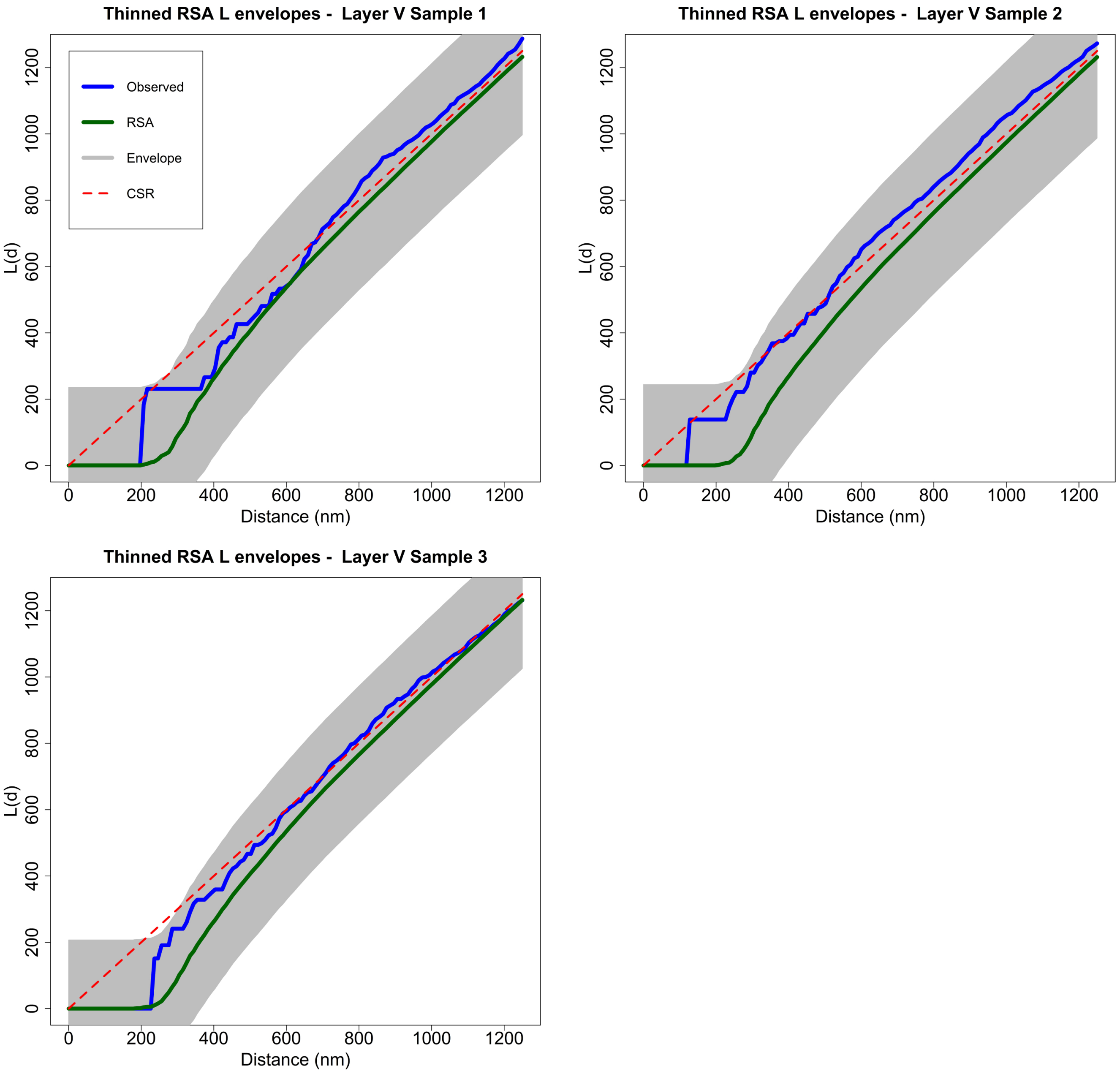

Supplementary Figure 14. Samples of Layer V

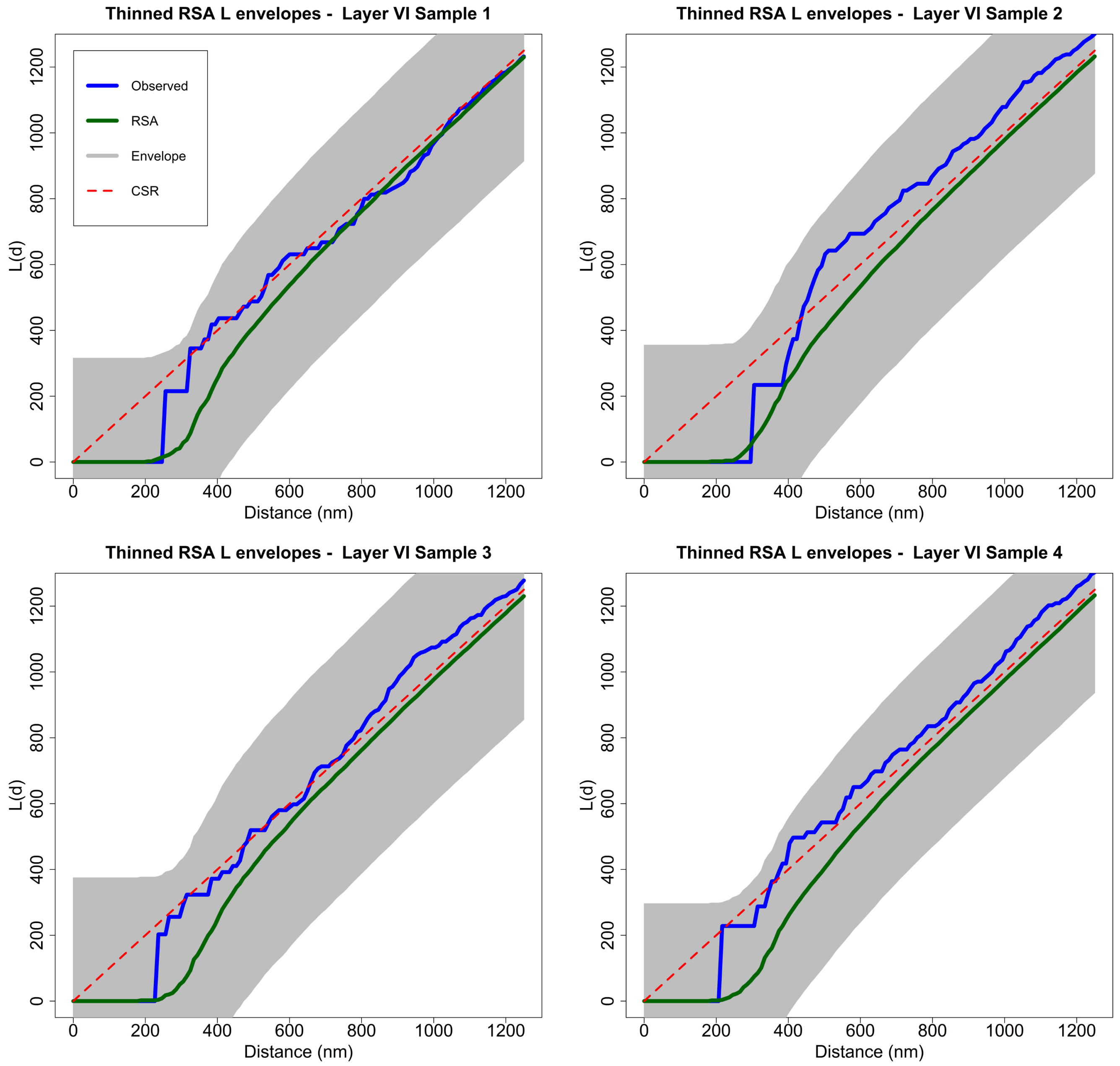

Supplementary Figure 15. Samples of Layer VI
